# Environmental reservoirs and mechanisms of persistence of *Vibrio cholerae*

**DOI:** 10.3389/fmicb.2013.00375

**Published:** 2013-12-16

**Authors:** Carla Lutz, Martina Erken, Parisa Noorian, Shuyang Sun, Diane McDougald

**Affiliations:** ^1^Centre for Marine Bio-Innovation, School of Biotechnology and Biomolecular Science, University of New South WalesSydney, NSW, Australia; ^2^Advanced Environmental Biotechnology Centre, Nanyang Environment and Water Research Institute, School of Biological Sciences, Nanyang Technological UniversitySingapore, Singapore; ^3^The Singapore Centre on Environmental Life Sciences Engineering, Nanyang Technological UniversitySingapore, Singapore

**Keywords:** starvation adaptation, biofilms, chitin, zooplankton, protozoa, predation, stress, viable but non-culturable

## Abstract

It is now well accepted that *Vibrio cholerae,* the causative agent of the water-borne disease cholera, is acquired from environmental sources where it persists between outbreaks of the disease. Recent advances in molecular technology have demonstrated that this bacterium can be detected in areas where it has not previously been isolated, indicating a much broader, global distribution of this bacterium outside of endemic regions. The environmental persistence of *V. cholerae* in the aquatic environment can be attributed to multiple intra- and interspecific strategies such as responsive gene regulation and biofilm formation on biotic and abiotic surfaces, as well as interactions with a multitude of other organisms. This review will discuss some of the mechanisms that enable the persistence of this bacterium in the environment. In particular, we will discuss how *V. cholerae* can survive stressors such as starvation, temperature, and salinity fluctuations as well as how the organism persists under constant predation by heterotrophic protists.

## INTRODUCTION

While it is likely to have been responsible for human infections and mortality throughout human history, cholera outbreaks have only been formally known to science since 1817 (Pollitzer, 1954). Sir John Snow was credited in 1849 as being the first person to connect contaminated water with cholera outbreaks and to use that information as an infection control strategy (Snow, 1855). In addition to being the genesis of modern epidemiology, his observation may also be the first study on the ecology of *Vibrio cholerae*. However, it took another 120 years for *V. cholerae* to be recognized as an autochthonous aquatic bacterium rather than a human pathogen that is a transient resident of the aquatic environment (Colwell et al., 1977). *V. cholerae* has over 200 serogroups, with O1 and O139 being the causative agents of cholera, due to their carriage of the genes encoding cholera toxin (CT) and the toxin co-regulated pilus (TCP; Chatterjee et al., 2007). Surveys performed in non-endemic areas have shown that the majority of *V. cholerae* strains isolated are non-toxigenic (Faruque et al., 2004; Haley et al., 2012; Islam et al., 2013), which suggests that associations with the human host is only one small aspect of the *V. cholerae* life cycle and is not necessary for environmental persistence.

**FIGURE 1:**
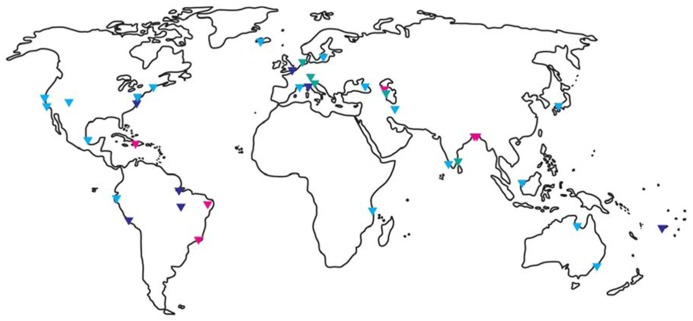
**Global distribution of *Vibrio cholerae*.** Triangles indicate where *V. cholerae* was detected by molecular and/or culture-based methods. Red indicates O1/O139 detection, light blue non-O1/non-O139 detection, and dark blue did not specify. Referenced studies here are only a small fraction of the studies published for certain areas and should guide as an example. North – and Middle America: (Colwell et al., 1981; Ogg et al., 1989; Blackwell and Oliver, 2008; Lizárraga-Partida et al., 2009; Hill et al., 2011; Dickinson et al., 2013), South America: (Franco et al., 1997; Lipp et al., 2003; Leal et al., 2008; Martinelli Filho et al., 2010; Sá et al., 2012); Africa: (Taviani et al., 2008); Europe: (Andersson and Ekdahl, 2006; Covazzi Harriague et al., 2008; Kirschner et al., 2008; Vezzulli et al., 2009, Vezzulli et al., 2011; Böer et al., 2013; Cantet et al., 2013; Tall et al., 2013); Middle East: (Bakhshi et al., 2009; Grim et al., 2010; Gurbanov et al., 2011; Rashid et al., 2013); Asia Pacific: (Islam et al., 1994, 2013; Desmarchelier et al., 1995; Miyagi et al., 2003; Alam et al., 2006; Vimala et al., 2010; de Magny et al., 2011; Singh et al., 2012).

*Vibrio cholerae* inhabits a vast geographical range from the tropics (e.g., the Bay of Bengal where pandemics still occur, e.g., Albert et al., 1993; Huq et al., 2005; de Magny et al., 2011) to temperate waters world-wide (e.g., USA, South America, Australia, Sweden, and Italy, e.g., Vezzulli et al., 2009; Collin and Rehnstam-Holm, 2011; Schuster et al., 2011; Islam et al., 2013; Tall et al., 2013; **Figure 1**). An increasing understanding of the ecology of *V. cholerae*, along with advances in molecular detection has demonstrated that this bacterium is a cosmopolitan aquatic species that is capable of causing illness in humans (Sack et al., 2004).

The capability to survive in many different environmental niches is largely due to the evolution of a range of adaptive responses that allow *V. cholerae* to survive stressors such as nutrient deprivation, fluctuations in salinity and temperature and to resist predation by heterotrophic protists and bacteriophage. One such strategy is the conversion into a viable but non-culturable (VBNC) state during unfavorable conditions (Colwell, 2000; Thomas et al., 2006). Additionally, *V. cholerae* attaches to abiotic and biotic surfaces (chitinous as well as gelatinous zoo- and phytoplankton) as biofilms (e.g., Huq et al., 1996; Akselman et al., 2010; Shikuma and Hadfield, 2010). Biofilm formation is associated with increased stress resistance, increased access to nutrients and as a means of dispersal when attached to living, mobile hosts (Costerton et al., 1995; Hall-Stoodley et al., 2004). Here, the current understanding of how *V. cholerae* is able to adapt to, and persist in the aquatic environment is summarized.

## SURFACE COLONIZATION AND BIOFILM FORMATION ENHANCE *V. cholerae* PERSISTENCE

**FIGURE 2:**
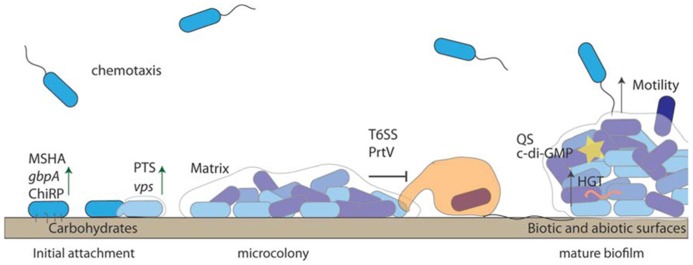
**Biofilm formation enhances *Vibrio cholerae* persistence.** *V. cholerae* uses chemotaxis to detect suitable surfaces for attachment. Substrate components, such as sugar concentrations of the conditioning film, play a role in the reversibly attached cells “decision” to become permanently attached. Permanent attachment is mediated by pili (ChiRP and MSHA) and outer membrane proteins such as GbpA. Biofilm formation is re-enforced through the production of VPS, which is controlled by QS (yellow star) and c-di-GMP. Anti-protozoal mechanisms such as T6SS protect surface attached bacteria. *V. cholerae* within biofilms undergo horizontal gene transfer (HGT), which may aid in survival.

For aquatic bacteria, surface attachment provides a selective advantage through access to nutrients that accumulate at the liquid–surface interface (Dawson et al., 1981). Therefore, surface adhesion may be a survival strategy that allows bacteria to persist in nutrient-limited natural environments (Dawson et al., 1981; **Figure 2**). Additionally, some biotic surfaces may provide nutrients for attached bacteria (e.g., chitin; Nalin et al., 1979). Thus, it is not surprising that *V. cholerae* has been detected on many abiotic and biotic surfaces, including ship hulls (Shikuma and Hadfield, 2010), zooplankton (Tamplin et al., 1990; Epstein, 1993; Huq et al., 2005; Turner et al., 2009), macroalgae (Hood and Winter, 1997), and as floating aggregates (Alam et al., 2006).

*Vibrio cholerae* attachment is mediated by pili, which are surface expressed proteins, comprised of pilin subunits that promote surface attachment and subsequent biofilm formation. The ability of *V. cholerae* to attach to a range of surfaces is reflected in the variation in pilin subunits, and hence variation in pili, expressed by *V. cholerae* (Boyd and Waldor, 2002; Aagesen and Häse, 2012). One ecologically important substratum is chitin, and *V. cholerae*, as are most Vibrionaceae, is chitinolytic and possesses multiple conserved genes to attach to and degrade chitin (Meibom et al., 2004; Hunt et al., 2008). This organic polymer of *N*-acetylglucosamine (GlcNAc/NAG) is the second most abundant organic polymer in nature and is an excellent carbon source for bacteria (Rinaudo, 2006; Martínez et al., 2009). The binding of *V. cholerae* to chitin involves the GlcNAc binding protein, GbpA (Kirn et al., 2005; Stauder et al., 2010), as well as the mannose sensitive hemagglutinin (MSHA), which is a type IV pilus (Chiavelli et al., 2001; **Figure 2**). Furthermore, the TCP, which is a colonization factor of human intestinal epithelia, has a duel role in association with chitin. TCP is required for differentiation of attached biofilms, and undifferentiated biofilms lacking TCP have reduced ecological fitness, as they are less efficient at degrading chitin (Reguera and Kolter, 2005).

After initial surface attachment, *V. cholerae* forms “matrix-enclosed, surface-associated communities” or biofilms (Yildiz and Visick, 2009). *V. cholerae* biofilm formation is enhanced through the actions of type IV pili, flagella and production of the biofilm matrix, Vibrio polysaccharide (VPS; Watnick and Kolter, 1999). VPS is involved in cell immobilization, microcolony formation, and biofilm maturation (Watnick and Kolter, 1999; Watnick et al., 2001). High and low VPS producing *V. cholerae* colony types are referred to as “rugose” and “smooth,” respectively, with the rugose having a higher protective effect toward a variety of stresses, including chlorine (Rice et al., 1992; Morris et al., 1996; Yildiz and Schoolnik, 1999), low pH (Zhu and Mekalanos, 2003), osmotic and oxidative stress (Wai et al., 1998), anti-bacterial serum (Morris et al., 1996), SDS (Fong et al., 2006), phage (Nesper et al., 2001), and heterotrophic protists (Sun et al., 2013). The importance of VPS for protection in the environment is still unknown as there are few published reports on the occurrence of rugose *V. cholerae* in the environment (Ali et al., 2002; Jubair et al., 2012).

The structural genes for VPS production are encoded on two carbohydrate biosynthesis operons located on the large chromosome, which encodes many essential housekeeping genes (Yildiz and Schoolnik, 1999; Fong et al., 2010). The *vps*I operon contains the genes *vpsA* to *vpsK* and the *vps*II operon contains the genes *vpsL* to *vpsQ*. The six genes located between the two *vps* operons (*rbmA*–*F*) are also involved in biofilm formation (Fong and Yildiz, 2007; Absalon et al., 2011; Berk et al., 2012). The requirement for sugars in the synthesis of VPS is an important determinant for the control of biofilm formation (discussed in Section “*V. cholerae* Responses to Environmental Stresses ***–*** Bottom-up Control of *V. cholerae*”). In addition to sugars, multiple regulators control the expression of VPS. For example VPS biosynthesis is positively regulated by VpsR (Yildiz et al., 2001) and VpsT (Casper-Lindley and Yildiz, 2004) in a c-di-GMP-dependent manner (Krasteva et al., 2010; Srivastava et al., 2011). C-di-GMP is an intracellular secondary messenger that controls the surface association of bacteria in response to environmental conditions (Yildiz, 2008).

Bacterial cell–cell communication, or quorum sensing (QS), is critical for biofilm maturation and subsequent dispersal (Liu et al., 2007; Muller et al., 2007). At high *V. cholerae* cell densities, the QS response regulator, HapR, positively regulates the transcription of *hapA* encoding the hemagglutinin protease (HAP; Jobling and Holmes, 1997; Zhu et al., 2002), *cytR*, a repressor of biofilm development, flagellum biosynthesis genes (Yildiz et al., 2004), and represses VPS production and *toxR*, the regulator of virulence (Jobling and Holmes, 1997; Zhu et al., 2002; Hammer and Bassler, 2003; Zhu and Mekalanos, 2003; Yildiz et al., 2004). It is proposed that the coordination of QS and c-di-GMP controlled traits allows for survival through rapid adaptation to environmental conditions. For example, the switch from a free-swimming to an attached lifestyle (Yildiz and Visick, 2009; Srivastava and Waters, 2012) enables natural competency and horizontal gene transfer (HGT; Lo Scrudato and Blokesch, 2012) as well as provides enhanced predation resistance (Matz et al., 2005). Mechanisms such as biofilm formation enable the persistence of *V. cholerae* and are not limited to interactions with nutritive biotic factors. Indeed, as described in the following sections, many abiotic factors including temperature, salinity, and pH influence the expression of adaptive traits.

## “VIABLE BUT NON-CULTURABLE” *V. cholerae* IN PLANKTON

In contrast to starved cells, VBNC cells fail to grow on culture media normally used to grow them, and are often reduced in size but remain metabolically active (Nilsson et al., 1991; McDougald et al., 1998; Oliver, 2010). Since the discovery that *V. cholerae* can enter the VBNC state (Xu et al., 1982), many bacteria, pathogens as well as non-pathogens, have been shown to enter the VBNC state under unfavorable conditions (McDougald et al., 1998, 1999; Oliver, 2005, 2010). Factors known to induce VBNC formation in *V. cholerae* include extremes in temperature and salinity as well as nutrient deprivation (Colwell et al., 1985; Ravel et al., 1995; Carroll et al., 2001; González-Escalona et al., 2006; Thomas et al., 2006; Mishra et al., 2012). VBNC cells of *V. cholerae* have been detected on the surface of higher organisms, such as crustaceans and algae in the plankton and benthos, attached to chironomid egg masses, as well as suspended in bacterioplankton (e.g., Louis et al., 2003; Binsztein et al., 2004; Alam et al., 2007; Halpern et al., 2007). Interestingly, *V. cholerae* appears predominately as VBNC cells within the bacterioplankton and as culturable cells in biofilm consortia, either as aggregates or attached to biotic and abiotic surfaces (Alam et al., 2006). The importance of the VBNC state in cholera epidemiology was demonstrated by Mishra et al. (2012), where virulence and colonization traits were actively expressed in VBNC *V. cholerae* incubated in freshwater microcosms.

For the VBNC response to impart protection allowing for persistence during unfavorable conditions, the cells must be able to resuscitate and divide when conditions become favorable (McDougald et al., 1998). For example, *Vibrio vulnificus* enters the VBNC state and can be resuscitated when incubated in environmental diffusion chambers in the marine environment (Oliver et al., 1995). Just as there are numerous conditions that induce VBNC formation in different species, there are numerous factors that induce resuscitation, including temperature upshift (Nilsson et al., 1991; Mishra et al., 2012) or an increase in nutrients (Binsztein et al., 2004; Senoh et al., 2010).

VBNC *V. cholerae* cells have also been shown to regain culturability by passage through animal digestive tracts (Colwell et al., 1985; Alam et al., 2007; Asakura et al., 2007). Furthermore, the ingestion by human volunteers of *V. cholerae* cells that had been VBNC for as long as 7 weeks resulted in colonization of the intestine and excretion of culturable cells (Colwell et al., 1996). Thus, VBNC cells represent an important environmental reservoir of *V. cholerae* as an agent of disease. However, VBNC cells remain capable of resuscitation for a limited time, and eventually, these cells lose the ability to resuscitate (Weichart et al., 1997). For example, VBNC cells can be resuscitated after co-incubation with eukaryotic cell lines, but resuscitation does not occur for cells that have been VBNC for a prolonged time (more than 91 days; Senoh et al., 2010).

Recently, QS has been implicated in the regulation of the VBNC state. For example, transition of culturable *V. cholerae* to the VBNC state involves biofilm formation and was shown to be dependent on QS (Kamruzzaman et al., 2010). In accordance with these results, VBNC cells from surface waters in Bangladesh have been resuscitated by natural or chemically synthesized QS autoinducers, as high colony forming unit (CFU) counts were detected after 4–5 h of exposure to two different autoinducers (Bari et al., 2013).

One hypothesis for the non-culturability of viable cells on common agar plates is that accumulation of reactive oxygen species (ROS) in the non-growing VBNC cells is detrimental when growth is initiated after nutrient addition. Thus, increased nutrient could lead to an imbalance in metabolism resulting in the production of ROS and cell death (Bloomfield et al., 1998). In fact, treatment of VBNC *Escherichia coli* with catalase or peroxide-degrading compounds can restore culturability (Mizunoe et al., 1999) and elimination of hydrogen peroxide from starved cultures of *E. coli* can prevent VBNC formation (Arana et al., 1992). Furthermore, loss of culturability of *V. vulnificus* under low temperature incubation has been correlated with loss of catalase activity, making the cells ROS sensitive (Kong et al., 2004).

It was recently hypothesized that VBNC cells resuscitate in a stochastic manner rather than in response to environmental parameters (Epstein, 2009). The authors argue that some cells of a dormant community will randomly revive from dormancy and if conditions are favorable, they will grow. Thus these revived cells can be likened to “scouts” inspecting environmental conditions (Buerger et al., 2012a,b). If conditions are not permissive for growth, the scouts will die, resulting in the loss of only a small fraction of the population. However, if conditions are favorable, then the genetic pool is amplified and maintained. The authors demonstrated that sampled marine and soil bacteria randomly became culturable during long term incubation in the wells of microtiter plates containing single cells. Furthermore, strains that were slow growing initially, with a cultivation time of 3–4 weeks could be sub-cultured within 48–72 h (Buerger et al., 2012b). In this way, the VBNC state represents a low cost population-based strategy that allows bacteria to remain dormant in the environment for extended periods, and to potentially either revive when an appropriate cue is present, e.g., an inducing signal, or to randomly test their environment to subsequently grow when conditions are favorable. Although stochastic VBNC resuscitation was not tested with *V. cholerae*, it has implications for identifying resuscitation cues and for understanding triggers of *V. cholerae* growth and cholera outbreaks.

## *Vibrio cholerae* RESPONSES TO ENVIRONMENTAL STRESSES – BOTTOM-UP CONTROL OF *V. cholerae*

The occurrence of *Vibrio* spp. in the environment is correlated with temperature, salinity, and phyto- as well as zooplankton (Turner et al., 2009, 2013; Johnson et al., 2010; Asplund et al., 2011). High water temperature is a strong predictor for the presence of *Vibrio* spp. (Blackwell and Oliver, 2008; Lama et al., 2011; Johnson et al., 2012), as they are mainly detected in warmer waters (above 15°C). Many studies have demonstrated that the abundance of *Vibrio* spp*.* follows a seasonal pattern that is dictated to a large extent by temperature (e.g., Louis et al., 2003; Binsztein et al., 2004). Increased temperature can influence the attachment of *V. cholerae* to chitinous zooplankton. At temperatures above 15°C, attachment to chitin increases significantly due to an increase in the expression of the MSHA pilus and the colonization factor, GbpA (Castro-Rosas and Escartìn, 2005; Turner et al., 2009; Stauder et al., 2010). In contrast, despite the water temperatures in the Chesapeake Bay being above 19°C, *V. cholerae* was found more often in the water column, as planktonic cells, than attached to plankton (Louis et al., 2003). Thus, in addition to elevated temperature, other factors must influence biofilm formation or dispersal, demonstrating the importance of environmental surveying, collecting, and interpreting metadata to determine those factors that influence cholera epidemics.

Temperature fluctuations due to seasonal changes, as well as freshwater influx can strongly influence the salinity of marine water bodies. Open ocean waters have an average salinity of 35 ppt. However, near coastal and estuarine areas the salinity can drop due to freshwater input from rivers or rain run-off (Jutla et al., 2011), and can increase in areas with higher solar evaporation, especially in the tropics. *Vibrio* spp. grow preferably at salinities <25 ppt (e.g., Jiang, 2001; Thomas et al., 2006; Baker-Austin et al., 2010). In high salinity environments *V. cholerae* increases the production of the protective pigment, melanin (Coyne and al-Harthi, 1992), which provides UV resistance (Valeru et al., 2009). The relationship between *V. cholerae* occurrence and salinity appears to be variable, with some studies reporting a significant correlation (Singleton et al., 1982; Johnson et al., 2010), while others demonstrate a lack of correlation between the occurrence of the organism and salinity (Johnson et al., 2012). For example, Stauder et al. (2010) showed that different salinities had no effect on attachment to surfaces, which is important for environmental persistence (as discussed in Section “ Association with Other Organisms”).

Seasonal fluctuations are often correlated with changing nutrient concentrations, as rain run-off is generally higher in spring/autumn and in coastal and estuarine areas. This can lead to higher phytoplankton abundance, followed by zooplankton blooms (e.g., Lobitz et al., 2000; Huq et al., 2005), which provide the chitinous surfaces that harbor bacteria such as *V. cholerae*. This may enable overall numbers of the organism to increase in the environment even though bacterivorous predators are also more abundant.

Nutrient sources in the environment are not uniformly distributed but occur as microscale patches, influenced by localized events such as cell lysis and waste excretion (Blackburn et al., 1998). Planktonic bacteria use motility and chemotaxis to take advantage of such nutrient patches (for a review of see, Stocker and Seymour, 2012). *V. cholerae* possesses a single sheathed polar flagella (Hranitzky et al., 1980) powered by sodium motive force (Kojima et al., 1999). The number of duplicated chemotaxis-related genes possessed by *V*. *cholerae* indicates the importance of this response for environmental survival (Heidelberg et al., 2000). *V. cholerae* have multiple chemotaxis genes, however not all are required for chemotaxis under standard laboratory conditions, suggesting that the other genes act as accessory chemotactic genes or have as yet undiscovered functions in the environment (Gosink et al., 2002). *V. cholerae* has been shown to be chemotactic toward all amino acids (Freter and O’Brien, 1981), suggesting that proteins, peptides, or amino acids may be important nutrient sources in the aquatic environment. In addition, *V. cholerae* upregulates chemotaxis genes in response to chitin oligosaccharides, facilitating detection and attachment to chitinous organisms (Meibom et al., 2004).

The ability of *V. cholerae* to persist in the environment is intrinsically linked to biofilm formation and VPS synthesis, both of which allow for the exploitation of nutrients available at the surface. Concentrations of sugars, phosphorus, and nitrogen influence attachment and biofilm formation *V. cholerae* cells. The presence of glucose and mannose induce VPS synthesis during biofilm development (Kierek and Watnick, 2003; Moorthy and Watnick, 2004). The phosphoenolpyruvate phosphotransferase system (PTS) is one of the major sugar transport systems in *V. cholerae* and activation of PTS results in derepression of VPS gene transcription and thus increased biofilm formation (Houot and Watnick, 2008; Houot et al., 2010). In addition, a *V. cholerae* PTS that responds to intracellular nitrogen concentrations, is believed to repress VPS production, however the receptor molecule and signaling pathway are still unknown (Houot et al., 2010).

Phosphorous also affects surface colonization. In phosphorus depleted environments, *V. cholerae* adopts a free-swimming planktonic lifestyle that is mediated by a two-component system, PhoBR. The histidine kinase, PhoR, phosphorylates the response regulator, PhoB, resulting in further repression of VPS production (Pratt et al., 2009; Sultan et al., 2010).

Planktonic *V. cholerae* cells have been shown to settle in response to extracellular DNA (eDNA), which is a component of the pre-established biofilm matrix (Haugo and Watnick, 2002). This occurs by repression of CytR, which in turn represses VPS and biofilm formation (Haugo and Watnick, 2002). Since *V. cholerae* is rich in DNases (Focareta and Manning, 1991), the eDNA maybe utilized as a nutrient source (Seper et al., 2011).

Since nutrient availability fluctuates in the aquatic environment, the ability to store essential nutrients is an important trait for bacteria that live a “feast-to-famine lifestyle.” In bacteria, glycogen is stored in granules and can serve as a carbon source during periods of starvation (Preiss and Romeo, 1994). Under nutrient rich conditions *V. cholerae* increases glycogen storage precursors (Kan et al., 2004). In addition, glycogen granules are present in nutrient poor rice water stools shed by patients with cholera (Bourassa and Camilli, 2009), indicating that glycogen storage may provide nutrients to *V. cholerae* as it passages from the human host into the aquatic environment. In addition to glycogen storage, the ability to store inorganic phosphorus (Pi) facilitates protection against environmental stresses such as acidity, salinity fluctuations, and the damaging effects of hydrogen peroxide, as it is required for activity of the general stress response regulator, RpoS (Jahid et al., 2006). *V. cholerae* is also able to store Pi within membrane bound granules at 100 times the concentrations achieved by *E. coli* (Ogawa et al., 2000). *V. cholerae* mutants deficient in Pi storage displayed reduced attachment to abiotic surfaces, decreased motility and a delayed adaptation to high calcium media (200 mM) (Ogawa et al., 2000).

In addition to carbon and phosphorous, iron is also a growth limiting factor required for cellular metabolism as it is a component of many cofactors (Wackett et al., 1989) and has low solubility in aquatic environments (Martin, 1992). Iron concentrations in the aquatic environment are highly variable and are generally correlated with water depth (Martin and Michael Gordon, 1988). *V. cholerae* has evolved several iron transport systems and receptors that enable persistence in low iron environments (Heidelberg et al., 2000; Wyckoff et al., 2006, 2007). These iron acquisition systems include a catechol siderophore, vibriobactin (Griffiths et al., 1984), and a transport system, Feo, that can take up ferrous iron (Wyckoff et al., 2006). Most iron acquisition genes, such as *irgA* (Goldberg et al., 1991), are repressed by the ferric uptake regulator (Fur) in environments with sufficient iron, as Fe(II)-Fur binds to the promoter of iron-regulated genes, preventing their expression (Bagg and Neilands, 1987). *V. cholerae* can also make use of siderophores secreted by other microorganisms, such as fluvibactin synthesized by *Vibrio fluvialis*, as it possesses the required receptors and uptake systems (Yamamoto et al., 1993).

In nutrient limited environments, *V. cholerae* can enter a starvation state, in which the cells are non-growing but culturable. In a recent laboratory study, Jubair et al. (2012) described the long-term starvation survival of *V. cholerae* (700 days). The authors suggest the term “persister phenotype” to differentiate starved cells from the VBNC state. The growth of persister cells was enhanced in the presence of phosphate and chitin, both important nutrients, which further highlights their importance for *V. cholerae* survival. An earlier study on the behavior of *V. cholerae* starved for 40 days showed that chitin attachment ligands were maintained (Pruzzo et al., 2003). These findings demonstrate the importance of association with chitinous organisms with details of specific interactions discussed in the Section “Association with Other Organisms.”

## TOP-DOWN CONTROL BY PREDATORY MICROGRAZERS

While availability of nutrients exerts “bottom-up” control of *V. cholerae*, predation by heterotrophic protists is one of the major mortality factors faced by bacteria in the environment (Hahn and Höfle, 2001; Matz and Kjelleberg, 2005). As part of the bacterioplankton, *V. cholerae* is under constant predation pressure by phagotrophic protists and other bacterivorous members of the zooplankton community. The long-term persistence and seasonal accumulation of *V. cholerae* is dependent on how it responds to this stress. Microcosm studies of natural bacterioplankton communities from the Gulf of Mexico showed that ciliates and heterotrophic nanoflagellates (HNFs) efficiently eliminate *V. cholerae* from environmental water samples (Martínez Pérez et al., 2004). In addition, ciliates as well as flagellates can feed on *V. cholerae*, with grazing rates of up to 600–2,000 bacteria cell^-1^ h^-1^ (Macek et al., 1997). Control of *V. cholerae* numbers by heterotrophic protists was also demonstrated by Worden et al. (2006), where *V. cholerae* growth rates of up to 2.5 doublings per day were countered by heavy grazing mortality by HNFs. During intense phytoplankton blooms, these growth rates increased to more than four doublings per day, allowing *V. cholerae* to overcome grazing pressure, potentially attaining sufficient numbers in the environment to reach an infectious dose.

*Vibrio cholerae* cells encased in a biofilm matrix are protected from predation by HNFs, while planktonic cells are rapidly eliminated (Matz et al., 2005). Predation induces biofilm formation and a smooth to rugose morphological shift, due to an increase in VPS production (Matz et al., 2005). VPS has subsequently been confirmed to be partly responsible for biofilm grazing resistance, where the *V. cholerae* cells encased in VPS were protected from predators (Sun et al., 2013). In addition to physical protection provided by biofilms, the high cell density in *V. cholerae* biofilms provides a sufficient quorum to promote expression of several QS-regulated anti-protozoal factors that cannot accumulate in the planktonic phase.

The importance of QS for protection from protozoal predation is supported by field tests demonstrating that QS-deficient *V. cholerae* was more susceptible to grazing than the wild type. However, the QS mutant strain did not lose all grazing resistance, suggesting that *V. cholerae* regulates anti-protozoal activities by a combination of QS and other regulatory systems (Erken et al., 2011). VPS mutants were less resistant than the wild type strain to surface grazing by the amoeba, *Acanthamoeba castellanii* and the HNF, *Rhynchomonas nasuta*, but were more resistant than the *hapR* mutant strain, indicating that QS is more protective than VPS against predators (Sun et al., 2013). QS has been shown to regulate secreted compounds that provide resistance from functionally different predators such as *Tetrahymena pyriformis, Cafeteria roenbergensis,* and *Caenorhabditis elegans*, e.g., an uncharacterized anti-protozoal factor (Matz et al., 2005) and the PrtV protease (Vaitkevicius et al., 2006).

The type VI secretion system (T6SS) also functions as an anti-predation mechanism that is inhibitory against *Dictyostelium discoideum*, mammalian macrophages, and *E. coli* (Pukatzki et al., 2006; MacIntyre et al., 2010). Three proteins (VgrG-1, -2, and -3) secreted by the T6SS form syringe-like structures, puncturing the cell membrane and delivering a virulence factor, VasX, into *D. discoideum* (Pukatzki et al., 2007; Miyata et al., 2011). The expression of another major component of T6SS, Hcp, is positively regulated by QS in *V. cholerae* (Ishikawa et al., 2009). Although all *V. cholerae* strains have this system, expression differs between them (Unterweger et al., 2012). For example, pandemic El Tor strains do not express T6SS under laboratory conditions while in some non-O1/non-O139 strains T6SS is constitutively expressed (Miyata et al., 2010).

Another mechanism for surviving protozoan predation is the ability of the bacterium to survive digestion. Both clinical and environmental strains of *V. cholerae* can survive intracellularly in a range of amoeba (Abd et al., 2004, 2005; Jain et al., 2006). Several studies have demonstrated that *V. cholerae* growth is enhanced when associated with free-living amoeba (Thom et al., 1992; Sandström et al., 2010; Valeru et al., 2012), further demonstrating the role amoeba play as environmental reservoirs of *V. cholerae*. In addition to surviving within amoebic trophozoites, *V. cholerae* cells have been found in the stress resistant cysts formed by amoeba, providing protection from environmental stresses (Thom et al., 1992; Abd et al., 2004), as well as a vehicle for dispersal throughout the aquatic environment (Thom et al., 1992; Brown and Barker, 1999). Thus, amoeba cysts could potentially facilitate the spread of cholera (Winiecka-Krusnell and Linder, 2001). Although many reports have characterized the relationship between *V. cholerae* and amoeba (Thom et al., 1992; Abd et al., 2005, 2007; Sandström et al., 2010), very little is known about the mechanisms that facilitate intracellular survival, although survival of the acidic conditions encountered within the digestive vacuoles has been attributed to an inducible acid tolerance response (Merrell and Camilli, 1999). In addition, ToxR has been shown to be important for survival in amoeba (Valeru et al., 2012). The authors suggest that it may be the ToxR-regulated outer membrane proteins, OmpU and OmpT that are responsible for enhanced survival. Experimentally, attraction and attachment to a protozoan host cell has yet to be shown (Abd et al., 2009, 2011). However, adhesins such as MSHA or capsule/LPS O side chain are not involved (Lock et al., 1987; Abd et al., 2009).

There is a lack of knowledge regarding the type and function of other virulence factors in facilitating intracellular survival in protozoa, especially when compared with other bacteria such as *Legionella* spp. and *Salmonella* spp. (Rowbotham, 1980; Bozue and Johnson, 1996; Brandl et al., 2005). There are many important questions that need to be addressed regarding *V. cholerae*–protozoa interactions, including how prevalent these interactions are in the environment and whether they facilitate resuscitation from the VBNC state. In addition, it is important to explore the prevalence of survival and passage through predatory protists and whether the bacterium remains viable in fecal pellets. A further understanding of the roles higher organisms play in the enhancement of *V. cholerae* fitness traits is required to understand the persistence and spread of the pathogen in the environment.

In addition to aforementioned predation pressure by phagotrophic protists, phage, and predatory bacteria also affect the abundance and serogroup prevalence of *V. cholerae* in the environment. For example, the CTXφ phage carries the genes encoding CT and is required for conversion of non-toxigenic to toxigenic strains (Miller and Mekalanos, 1988; Pearson et al., 1993; Waldor and Mekalanos, 1996). Phage predation has shaped cholera epidemics in Bangladesh, where high concentrations of phage are detected after an initial peak in cholera cases and numbers of *V. cholerae* cells in the aquatic environment (Faruque et al., 2005). Following the increase in phage numbers, the number of cholera cases decreases. An increase in phage numbers in the environment was also correlated with an increase in *V. cholerae* lytic bacteriophage in patient stool samples, with one of the predominant bacteriophage species belonging to the *Myoviridae* family (Seed et al., 2011). Environmental surveys have detected *Myoviridae* in regions where cholera outbreaks have occurred, such as Peru (Talledo et al., 2003), Kolkata (Sen and Ghosh, 2005), and Kenya (Maina et al., 2013). Control of *V. cholerae* by phage is supported by a continuous culture experiment, which suggests that *V. cholerae* populations may be influenced by phage to a larger extent than by nutrient limitation (Wei et al., 2011). Predatory bacteria such as *Bdellovibrio* sp. also prey on *V. cholerae* (Chen et al., 2012) and might also shape *V. cholerae* occurrence in the environment. However, there is limited information on the interactions between predatory bacteria and *V. cholerae*.

## ASSOCIATION WITH OTHER ORGANISMS

**FIGURE 3:**
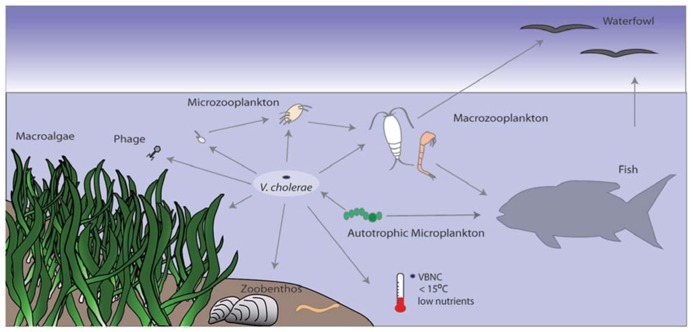
***Vibrio cholerae* interactions with other organisms and the environment.** *V. cholerae* is part of the bacterioplankton in aquatic environments. It is under predation pressure by protozoa and bacteriophage and is thus incorporated into the microbial loop. Low temperature and nutrient conditions can trigger the VBNC state, from which it resuscitates under more favorable conditions. *V. cholerae* can also attach to autotrophic organisms such as phytoplankton or macroalgae, which can provide a carbon source. Attachment to chitinous zooplankton and gelatinous egg masses (e.g., chironomids) provide nutrients and also facilitate HGT. Fish and birds feed on plankton or mussels that might harbor *V. cholerae* and can potentially spread the bacterium across long distances.

*Vibrio cholerae* is an integral part of the aquatic environment and in addition to heterotrophic protists, interacts with a wide range of organisms. It has been demonstrated to interact with water fowl (Halpern et al., 2008), fish (Kiiyukia et al., 1992; Senderovich et al., 2010), chironomids (Broza and Halpern, 2001; Halpern et al., 2006), mussels (Deriu et al., 2002; Collin et al., 2012), cyanobacteria (Islam et al., 1999), diatoms (Binsztein et al., 2004; Seeligmann et al., 2008), and dinoflagellates (Binsztein et al., 2004; Akselman et al., 2010; **Figure 3**). The association of *V. cholerae* with zooplankton has been a topic of study since the discovery of cells attached to the surface of copepods in the early 1980s (Huq et al., 1983; Tamplin et al., 1990). Zooplankton are an important part of the aquatic food web, grazing on autotrophic and heterotrophic bacterio-, nano-, and micro-plankton and are in turn preyed upon by larger plankton, such as insect and crustacean larvae and fish. One well-studied interaction is that between *V. cholerae* and chitinous zooplankton, e.g., copepods and cladocerans (Nalin et al., 1979; Huq et al., 1983; Rawlings et al., 2007). For example, pivotal experiments link the transmission of cholera with zooplankton (Huq et al., 1996, 2005; Colwell et al., 2003). In a now classic experiment, the filtration of water through readily available sari cloth reduced *V. cholerae* numbers by 99% (Huq et al., 1996). This method proved to be effective in field trials in reducing the incidence of cholera cases and was continued by villagers as a treatment for drinking water (Colwell et al., 2003; Huq et al., 2010). de Magny et al. (2011) suggested the use of different zooplankter to predict cholera epidemics as they demonstrated that the cladocerans, *Monia* spp. and *Diphanosoma* spp. as well as the rotifer *Brachionus angularis*, were significantly correlated with the presence of *V. cholerae* and with cholera outbreaks. *V. cholerae* has repeatedly been found to be associated with the copepod *Acartia tonsa,* which appears to harbor higher numbers of *V. cholerae* than co-occurring copepods (e.g., Huq et al., 1983; Binsztein et al., 2004; Rawlings et al., 2007; Lizárraga-Partida et al., 2009, for further information, see Pruzzo et al., 2008).

The predominantly attached lifestyle of *V. cholerae* enables it to use many different biotic surfaces as nutrient sources. In addition to degrading chitin, *V. cholerae* has the ability to degrade the egg masses of chironomids (Broza and Halpern, 2001; Halpern et al., 2004). The production of the QS-regulated HAP is necessary for the degradation of the gelatinous matrix of the egg masses (Halpern et al., 2003). Although high numbers of *V. cholerae* were found attached to the egg masses (3.9 × 10^4^ per egg mass; Halpern et al., 2007) 99.5% of the attached cells were other species, e.g., *Acinetobacter, Aeromonas, Klebsiella, Shewanella,* and *Pseudomonas*. These species may benefit from the nutrients that are released by *V. cholerae* as it degrades the egg mass. Alternatively, these species may have a negative impact on *V. cholerae* by expressing bacteriocins or competing for nutrients and space, which may in part explain why the majority of *V. cholerae* on the egg masses, 99.7%, were VBNC (Halpern, 2011). *V. cholerae* has been shown to be associated with chironomids in all four stages of development, from egg to adult (Broza and Halpern, 2001; Halpern et al., 2003; Broza et al., 2005), suggesting these insect eggs and larvae can serve as vectors for the transmission of cholera. Indeed, chironomids that were collected in air 3 km away from a water source were found to harbor *V. cholerae* and thus, these midges can carry the pathogen from one body of water to another (Broza et al., 2005). Although no toxigenic serogroups of *V. cholerae* have been detected in association with chironomids to date, it remains possible that these could also be associated with chironomids (Halpern, 2011).

The association between *V. cholerae* and phytoplankton has been well studied (e.g., Tamplin et al., 1990; Lobitz et al., 2000; Turner et al., 2009). Autotrophic protists, such as diatoms and dinoflagellates (Binsztein et al., 2004; Eiler et al., 2006), cyanobacteria (Islam et al., 1999; Eiler et al., 2007) as well as macroalgae (Vugia et al., 1997; Haley et al., 2012) support *V. cholerae* growth (e.g., Vezzulli et al., 2010). Various laboratory and environmental studies have shown that *V. cholerae* cells attach to microalgae cells. In a study off the coast of Argentina, Seeligmann et al. (2008) detected 1–10 VBNC *V. cholerae* cells attached to a single algal cell. It was suggested that attachment to phytoplankton might enable *V. cholerae* to survive prolonged exposure in freshwater environments due to the nutrients and salts excreted by the phytoplankton cells (Islam et al., 1989; Tamplin et al., 1990; Binsztein et al., 2004). Nutrients supplied by phytoplankton, e.g., due to a massive bloom, can also support explosive growth of *V. cholerae* (Mouriño-Pérez et al., 2003). In fact, remote sensing of chlorophyll *a* has been proposed as a method to predict cholera outbreaks (Lobitz et al., 2000).

Attachment of *V. cholerae* to macroalgae is induced by the plant-derived polyamine, norspermidine (Hamana and Matsuzaki, 1982). Norspermidine is bound by NspS, a periplasmic spermidine-binding protein that interacts with the periplasmic portion of the membrane protein, MbaA, resulting in induction of biofilm formation (Karatan et al., 2005). Mannitol, which is a product of photosynthesis by brown algae and secreted at high concentrations (Yamguchi et al., 1969; Ymele-Leki et al., 2013), induces *V. cholerae* settlement and VPS-dependent biofilm formation, resulting in transcription of *mtlA*, encoding a mannitol specific transport protein (Ymele-Leki et al., 2013). Once mannitol is transported into the *V. cholerae* cell it is possibly used as a carbon source or an osmoprotectant (Ymele-Leki et al., 2013).

*Vibrio cholerae* has also been detected in the gut of various species of fish that these feed on phyto- as well as zooplankton (Senderovich et al., 2010). *V. cholerae* numbers as high as 5 × 10^3^ cells per gram of intestine content were detected in fish sampled from different marine and freshwater environments in Israel (Senderovich et al., 2010). Non-O1 *V. cholerae* has also been detected in the kidneys, livers, and spleens of diseased, or homogenates of healthy ayu fish sampled from different rivers in Japan (Kiiyukia et al., 1992). Although no isolate carried CT genes, the supernatant of the cultures produced fluid accumulation in suckling mice. In addition to fish, waterfowl have been connected to the spread of *V. cholerae* (Ogg et al., 1989). Sea birds feed on zooplankton as well as phytoplankton and come in contact with these organisms by swimming on the water. Here, these planktonic organisms can then attach to bird feathers and thus *V. cholerae* can potentially be spread by air (Halpern et al., 2008).**

Most research on environmental *V. cholerae* has focused on the occurrence of the bacterium within the planktonic community or on the interactions of *V. cholerae* with planktonic organisms. However, recent research has shown that benthic communities also harbor high numbers of Vibrios, including *V. cholerae* (e.g., Covazzi Harriague et al., 2008; Vezzulli et al., 2009; Collin and Rehnstam-Holm, 2011). For example, bivalves are benthic filter feeders connecting the plankton and the benthos by feeding on the plankton. Bivalves such as mussels and oysters can harbor high numbers of pathogenic *Vibrio* spp. in their tissue and are an important niche for these bacteria (e.g., Olafsen et al., 1993; Maugeri et al., 2001; Kirs et al., 2011). Food poisoning resulting from the ingestion of contaminated raw or undercooked seafood is a major threat to human health. While infection by *V. vulnificus* and *Vibrio parahaemolyticus* from ingestion of seafood are the most common (Wright and Harwood, 2013), mussels can also harbor high numbers of *V. cholerae* and thus are a potential health threat (e.g., Murphree and Tamplin, 1995; Bauer et al., 2006; Haley et al., 2012).

The bivalve immune system consists of hemocytes (phagocytic cells) and the hemolymph (i.e., lysosomal enzymes and antimicrobial peptides; Mitta et al., 2000; Pruzzo et al., 2005). In order to reside in bivalve tissues, bacteria need to survive the antimicrobial activity of the hemolymph and engulfment by the hemocytes. Vibrios are resistant to depuration treatments (Murphree and Tamplin, 1995) and show resistance toward the hemocytes of the blue mussel *Mytilus edulis* (Hernroth et al., 2010). Some Vibrios were able to inhibit filtration in adult *M. edulis*, which was not correlated with the binding of the bacteria to the gills of the mussels (Birkbeck et al., 1987), suggesting another mechanism is involved. Interestingly *V. cholerae* strains of different origin have different retention times in mussels (Collin et al., 2012). An environmental *V. cholerae* strain isolated from the blue mussel was both taken up and eliminated much faster than a clinical non-O1/O139 strain. The clinical strain had a much longer retention time, implying that pathogenic strains have better fitness in the mussel than environmental strains. This has implications for control measures such as depuration, as they will be less effective at removing clinical strains than environmental strains. In addition, Collin et al. (2012) identified a highly virulent El Tor strain that was not ingested at all, indicating that bivalves did not eliminate this pathogenic strain from the water column. These results highlight the importance of interaction of *V. cholerae* with other organisms in its environment and the evolution and selection for virulent strains.

In addition to being incorporated into the benthos by filter feeders, *V. cholerae* can be isolated from sediments in numbers that are much higher than in the planktonic phase (Covazzi Harriague et al., 2008; Vezzulli et al., 2009). Sediments may therefore also act as a reservoir for cholera, especially in colder months, seeding the water column when temperatures rise (Vezzulli et al., 2009). Interestingly, in this study nematodes accounted for the highest abundance of the meiofauna, and bacterivorous nematodes accounted for 50% of the total. This suggests that *Vibrio* spp. are under high grazing pressure and top-down control by these nematodes (Vezzulli et al., 2009). In a laboratory study with *C. elegans*, Vaitkevicius et al. (2006) showed that *V. cholerae* kills the nematode after ingestion by secreting the extracellular protease PrtV. Neither CT nor TCP were required for the killing. Interestingly, PrtV was also required to prevent grazing by the flagellate *C. roenbergensis* and the ciliate *T. pyriformis*. In a Δ*hapR* strain, the ability to kill the nematode was strongly diminished. This is in accordance with the role of the QS response regulator, *hapR*, which is important for grazing resistance in the laboratory (Matz et al., 2005) as well as in the environment (Erken et al., 2011). Thus, *V. cholerae* has evolved or acquired a number of genetic systems that facilitate its ability to resist top-down control exerted by predatory eukaryotes.

## CONCLUSIONS

*Vibrio cholerae* is a significant pathogen that has played an important role in human history. Its role in the spread of disease and in epidemics has been reported for more than 150 years and the organism has even played an important role in establishment of modern epidemiology. While the mechanisms leading to infection and epidemics have been well studied, the ecology and mechanisms that underpin environmental persistence have been less well documented. Interestingly, environmental *V. cholerae* strains are largely represented by non-toxigenic strains and indeed, environmental strains display a high degree of genetic variability which has been suggested to aid in *V. cholerae* environmental stress resistance and subsequent persistence. The bacterium has an array of genetic systems involved in stress resistance, when faced with nutrient starvation, iron limitation, or changes in salinity and temperature. One such adaptation is the ability to grow as a biofilm on a range of abiotic and biotic surfaces. This not only increases resistance to stress, but may also directly provide access to nutrients, such as when attached to chitinous surfaces. Biofilm formation has also been directly linked to avoidance of predation by microeukaryotes. Predation resistance can be provided either by physical protection offered by the biofilm, the production of anti-predator compounds or defensive molecules or both. Perhaps not surprisingly, some of the gene systems involved in anti-predator defenses are the same as those associated with virulence during human infection. This may support the co-incidental virulence hypothesis that suggests that virulence factors evolve, at least in part, from the competition between predator and prey rather than against a human host. *V. cholerae* is a common inhabitant of many marine and freshwater habitats and this is most likely because it has evolved a range of strategies to enable its persistence in the natural environment. The identification and elucidation of these mechanisms, from ecological, evolutionary and molecular perspectives are likely to deliver exciting discoveries for the next 150 years.

## Conflict of Interest Statement

The authors declare that the research was conducted in the absence of any commercial or financial relationships that could be construed as a potential conflict of interest.

## References

[B1] Aagesen A Häse C. (2012). Sequence analyses of type IV pili from *Vibrio cholerae, Vibrio parahaemolyticus*, and *Vibrio vulnificus*. *Microb. Ecol.* 64 509–524 10.1007/s00248-012-0021-2 22383120

[B2] Abd H. Saeed A. Weintraub A. Nair G. B Sandström G. (2007). *Vibrio cholerae* O1 strains are facultative intracellular bacteria, able to survive and multiply symbiotically inside the aquatic free-living amoeba *Acanthamoeba castellanii*. *FEMS Microbiol. Ecol.* 60 33–39 10.1111/j.1574-6941.2006.00254.x 17381524

[B3] Abd H. Saeed A. Weintraub A Sandström G. (2009). *Vibrio cholerae* O139 requires neither capsule nor LPS O side chain to grow inside *Acanthamoeba castellanii*. *J. Med. Microbiol.* 58 125–131 10.1099/jmm.0.004721-0 19074664 PMC2884941

[B4] Abd H. Shanan S. Saeed A Sandström G. (2011). Survival of *Vibrio cholerae* inside *Acanthamoeba* and detection of both microorganisms from natural water samples may point out the amoeba as a protozoal host for V. *cholerae. J. Bacteriol. Parasitol.* 4 109

[B5] Abd H. Weintraub A Sandström G. (2004). Interaction between *Vibrio cholerae* and *Acanthamoeba castellanii*. *Microb. Ecol. Health Dis.* 16 51–57 10.1080/08910600410029190

[B6] Abd H. Weintraub A Sandström G. (2005). Intracellular survival and replication of *Vibrio cholerae* O139 in aquatic free-living amoebae. *Environ. Microbiol.* 7 1003–1008 10.1111/j.1462-2920.2005.00771.x 15946296

[B7] Absalon C. Van Dellen K. Watnick P. I. (2011). A communal bacterial adhesin anchors biofilm and bystander cells to surfaces. *PLoS Pathog.* 7:e1002210 10.1371/journal.ppat.1002210 PMC3161981 21901100

[B8] Akselman R. Jurquiza V. Costagliola M. C. Fraga S. G. Pichel M. Hozbor C. (2010). *Vibrio cholerae* O1 found attached to the dinoflagellate *Noctiluca scintillans* in Argentine shelf waters. *Mar. Biodivers. Rec.* 3 e120 10.1017/S1755267210001077

[B9] Alam M. Sultana M. Nair G. B. Sack R. B. Sack D. A. Siddique A. K. (2006). Toxigenic *Vibrio cholerae* in the aquatic environment of Mathbaria, Bangladesh. *Appl. Environ. Microbiol.* 72 2849–2855 10.1128/AEM.72.4.2849-2855.2006 16597991 PMC1449004

[B10] Alam M. Sultana M. Nair G. B. Siddique A. K. Hasan N. A. Sack R. B. (2007). Viable but nonculturable *Vibrio cholerae* O1 in biofilms in the aquatic environment and their role in cholera transmission. *Proc. Natl. Acad. Sci. U.S.A.* 104 17801–17806 10.1073/pnas.0705599104 17968017 PMC2077051

[B11] Albert M. Ansaruzzaman M. Bardhan P. Faruque A. Faruque S. Islam M. (1993). Large epidemic of cholera-like disease in Bangladesh caused by *Vibrio cholerae* 0139 synonym Bengal. *Lancet* 342 387–390 10.1016/0140-6736(93)92811-7 8101899

[B12] Ali A. Rashid M. H. Karaolis D. K. (2002). High-frequency rugose exopolysaccharide production by *Vibrio cholerae*. *Appl. Environ. Microbiol.* 68 5773–5778 10.1128/AEM.68.11.5773-5778.2002 12406780 PMC129946

[B13] Andersson Y. Ekdahl K. (2006). Wound infections due to *Vibrio cholerae* in Sweden after swimming in the Baltic Sea, summer 2006. *Euro. Surveill.* 11 E060803.2 10.2807/esw.11.31.03013-en 16966771

[B14] Arana I. Muela A. Iriberri J. Egea L. Barcina I. (1992). Role of hydrogen peroxide in loss of culturability mediated by visible light in *Escherichia coli* in a freshwater ecosystem. *Appl. Environ. Microbiol.* 58 3903–3907 1476433 10.1128/aem.58.12.3903-3907.1992 PMC183202

[B15] Asakura H. Ishiwa A. Arakawa E. Makino S. I. Okada Y. Yamamoto S. (2007). Gene expression profile of *Vibrio cholerae* in the cold stress-induced viable but non-culturable state. *Environ. Microbiol.* 9 869–879 10.1111/j.1462-2920.2006.01206.x 17359259

[B16] Asplund M. E. Rehnstam-Holm A. S. Atnur V. Raghunath P. Saravanan V. Härnström K. (2011). Water column dynamics of *Vibrio* in relation to phytoplankton community composition and environmental conditions in a tropical coastal area. *Environ. Microbiol.* 13 2738–2751 10.1111/j.1462-2920.2011.02545.x 21895909

[B17] Bagg A. Neilands J. B. (1987). Ferric uptake regulation protein acts as a repressor, employing iron (II) as a cofactor to bind the operator of an iron transport operon in *Escherichia coli*. *Biochemistry* 26 5471–5477 10.1021/bi00391a039 2823881

[B18] Baker-Austin C. Stockley L. Rangdale R. Martinez-Urtaza J. (2010). Environmental occurrence and clinical impact of *Vibrio vulnificus* and *Vibrio parahaemolyticus*: a European perspective. *Environ. Microbiol. Rep.* 2 7–18 10.1111/j.1758-2229.2009.00096.x 23765993

[B19] Bakhshi B. Barzelighi H. M. Adabi M. Lari A. R. Pourshafie M. (2009). A molecular survey on virulence associated genotypes of non-O1 non-O139 *Vibrio cholerae* in aquatic environment of Tehran, Iran. *Water Res.* 43 1441–1447 10.1016/j.watres.2008.12.025 19157484

[B20] Bari S. M. N. Roky M. K. Mohiuddin M. Kamruzzaman M. Mekalanos J. J. Faruque S. M. (2013). Quorum-sensing autoinducers resuscitate dormant *Vibrio cholerae* in environmental water samples. *Proc. Natl. Acad. Sci. U.S.A.* 110 9926–9931 10.1073/pnas.1307697110 23716683 PMC3683778

[B21] Bauer A. Ostensvik O. Florvag M. Ormen O. Rorvik L. M. (2006). Occurrence of *Vibrio parahaemolyticus*, V. *cholerae,* and *V. vulnificus* in Norwegian blue mussels(*Mytilus edulis*). *Appl. Environ. Microbiol.* 72 3058–3061 10.1128/AEM.72.4.3058-3061.2006 16598019 PMC1449022

[B22] Berk V. Fong J. C. N. Dempsey G. T. Develioglu O. N. Zhuang X. Liphardt J. (2012). Molecular architecture and assembly principles of *Vibrio cholerae* biofilms. *Science* 337 236–239 10.1126/science.1222981 22798614 PMC3513368

[B23] Binsztein N. Costagliola M. C. Pichel M. Jurquiza V. Ramiez F. C. Akselman R. (2004). Viable but nonculturable *Vibrio cholerae* O1 in the aquatic environment of Argentina. *Appl. Environ. Microbiol.* 70 7481–7486 10.1128/AEM.70.12.7481-7486.2004 15574951 PMC535145

[B24] Birkbeck T. McHenery J. Nottage A. (1987). Inhibition of filtration in bivalves by marine Vibrios. *Aquaculture* 67 247–248 10.1016/0044-8486(87)90045-7

[B25] Blackburn N. Fenchel T. Mitchell J. (1998). Microscale nutrient patches in planktonic habitats shown by chemotactic bacteria. *Science* 282 2254–2256 10.1126/science.282.5397.2254 9856947

[B26] Blackwell K. D. Oliver J. D. (2008). The ecology of *Vibrio vulnificus*, *Vibrio cholerae*, and *Vibrio parahaemolyticus* in North Carolina estuaries. *J. Microbiol.* 46 146–153 10.1007/s12275-007-0216-2 18545963

[B27] Bloomfield S. F. Stewart G. S. A. B. Dodd C. E. R. Booth I. R Power E. G. M. (1998). The viable but non-culturable phenomenon explained? *Microbiology* 144 1–3 10.1099/00221287-144-1-1 9467894

[B28] Bourassa L. Camilli A. (2009). Glycogen contributes to the environmental persistence and transmission of *Vibrio cholerae*. *Mol. Microbiol.* 72 124–138 10.1111/j.1365-2958.2009.06629.x 19226328 PMC2704980

[B29] Böer S. Heinemeyer E. A. Luden K. Erler R. Gerdts G. Janssen F. (2013). Temporal and spatial distribution patterns of potentially pathogenic *Vibrio* spp. at recreational beaches of the German North Sea. *Microb. Ecol.* 65 1052–1067 10.1007/s00248-013-0221-4 23563708

[B30] Boyd E. F. Waldor M. K. (2002). Evolutionary and functional analyses of variants of the toxin-coregulated pilus protein TcpA from toxigenic *Vibrio cholerae* non-O1/non-O139 serogroup isolates. *Microbiology* 148 1655–1666 12055286 10.1099/00221287-148-6-1655

[B31] Bozue J. A. Johnson W. (1996). Interaction of Legionella pneumophila with *Acanthamoeba castellanii*: uptake by coiling phagocytosis and inhibition of phagosome-lysosome fusion. *Infect. Immun.* 64 668–673 8550225 10.1128/iai.64.2.668-673.1996 PMC173819

[B32] Brandl M. T. Rosenthal B. M. Haxo A. F. Berk S. G. (2005). Enhanced survival of *Salmonella enterica* in vesicles released by a soilborne *Tetrahymena* species. *Appl. Environ. Microbiol.* 71 1562–1569 10.1128/AEM.71.3.1562-1569.2005 15746361 PMC1065168

[B33] Brown M. R. W. Barker J. (1999). Unexplored reservoirs of pathogenic bacteria: protozoa and biofilms. *Trends Microbiol.* 7 46–50 10.1016/S0966-842X(98)01425-5 10068997

[B34] Broza M. Gancz H. Halpern M. Kashi Y. (2005). Adult non-biting midges: possible windborne carriers of *Vibrio cholerae* non-O1 non-O139. *Environ. Microbiol.* 7 576–585 10.1111/j.1462-2920.2005.00745.x 15816934

[B35] Broza M. Halpern M. (2001). Pathogen reservoirs. Chironomid egg masses and *Vibrio cholerae. Nature* 412 40 10.1038/35083691 11452294

[B36] Buerger S. Spoering A. Gavrish E. Leslin C. Ling L. Epstein S. (2012a). Microbial scout hypothesis and microbial discovery. *Appl. Environ. Microbiol.* 78 3229–3233 10.1128/AEM.07308-11 22367084 PMC3346497

[B37] Buerger S. Spoering A. Gavrish E. Leslin C. Ling L. Epstein S. (2012b). Microbial scout hypothesis, stochastic exit from dormancy, and the nature of slow growers. *Appl. Environ. Microbiol.* 78 3221–3228 10.1128/AEM.07307-11 22367083 PMC3346438

[B38] Cantet F. Hervio-Heath D. Caro A. Le Mennec C. Monteil C. Quéméré C. (2013). Quantification of *Vibrio parahaemolyticus, Vibrio vulnificus* and *Vibrio cholerae* in French Mediterranean coastal lagoons. *Res. Microbiol.* 164 867–874 10.1016/j.resmic.2013.06.005 23770313 PMC4073583

[B39] Carroll J. Mateescu M. Chava K. Colwell R. R. Bej A. (2001). Response and tolerance of toxigenic *Vibro cholerae* O1 to cold temperatures. *Antonie Van Leeuwenhoek* 79 377–384 10.1023/A:1012004725373 11816983

[B40] Casper-Lindley C. Yildiz F. H. (2004). VpsT is a transcriptional regulator required for expression of vps biosynthesis genes and the development of rugose colonial morphology in *Vibrio cholerae* O1 El Tor. *J. Bacteriol.* 186 1574–1578 10.1128/JB.186.5.1574-1578.2004 14973043 PMC344397

[B41] Castro-Rosas J Escartìn E. F. (2005). Increased tolerance of *Vibrio cholerae* O1 to temperature, pH, or drying associated with colonization of shrimp carapaces. *Int. J. Food Microbiol.* 102 195–201 10.1016/j.ijfoodmicro.2004.12.015 15992618

[B42] Chatterjee A. Dutta P. K. Chowdhury R. (2007). Effect of fatty acids and cholesterol present in bile on expression of virulence factors and motility of *Vibrio cholerae*. *Infect. Immun.* 75 1946–1953 10.1128/IAI.01435-06 17261615 PMC1865667

[B43] Chen H. Young S. Berhane T. K. Williams H. N. (2012). Predatory Bacteriovorax communities ordered by various prey species. *PLoS ONE* 7:e34174 10.1371/journal.pone.0034174 PMC3312913 22461907

[B44] Chiavelli D. A. Marsh J. W. Taylor R. K. (2001). The mannose-sensitive hemagglutinin of *Vibrio cholerae* promotes adherence to zooplankton. *Appl. Environ. Microbiol.* 67 3220–3225 10.1128/AEM.67.7.3220-3225.2001 11425745 PMC93004

[B45] Collin B. Rehnstam-Holm A. S. (2011). Occurrence and potential pathogenesis of *Vibrio cholerae, Vibrio parahaemolyticus* and *Vibrio vulnificus* on the South Coast of Sweden. *FEMS Microbiol. Ecol.* 78 306–313 10.1111/j.1574-6941.2011.01157.x 21692819

[B46] Collin B. Rehnstam-Holm A. S. Lindmark B. Pal A. Wai S. N. Hernroth B. (2012). The origin of *Vibrio cholerae* influences uptake and persistence in the blue mussel *Mytilus edulis*. *J. Shellfish Res.* 31 87–92 10.2983/035.031.0111

[B47] Colwell R. R. (2000). Viable but nonculturable bacteria: a survival strategy. *J. Infect. Chemother.* 6 121–125 10.1007/PL00012151 11810550

[B48] Colwell R. R. Brayton P. Herrington D. Tall B. Huq A. Levine M. M. (1996). Viable but non-culturable *Vibrio cholerae* O1 revert to a cultivable state in the human intestine. *World J. Microbiol. Biotechnol.* 12 28–31 10.1007/BF00327795 24415083

[B49] Colwell R. R. Brayton P. R. Grimes D. J. Roszak D. B. Huq S. A. Palmer L. M. (1985). Viable but non-culturable *Vibrio cholerae* and related pathogens in the environment: implications for release of genetically engineered microorganisms. *Biotechnology* 3 817–820 10.1038/nbt0985-817

[B50] Colwell R. R. Huq A. Islam M. S. Aziz K. M. Yunus M. Khan N. H. (2003). Reduction of cholera in Bangladeshi villages by simple filtration. *Proc. Natl. Acad. Sci. U.S.A.* 100 1051–1055 10.1073/pnas.0237386100 12529505 PMC298724

[B51] Colwell R. R. Kaper J. Joseph S. (1977). *Vibrio cholerae, Vibrio parahaemolyticus*, and other vibrios: occurrence and distribution in Chesapeake Bay. *Science* 198 394–396 910135

[B52] Colwell R. R. Seidler R. J. Kaper J. Joseph S. W. Garges S. Lockman H. (1981). Occurrence of *Vibrio cholerae* serotype O1 in Maryland and Louisiana estuaries. *Appl. Environ. Microbiol.* 41 555–558 7235699 10.1128/aem.41.2.555-558.1981 PMC243732

[B53] Costerton J. W. Lewandowski Z. Caldwell D. E. Korber D. R. Lappin-Scott H. M. (1995). Microbial biofilms. *Annu. Rev. Microbiol.* 49 711–745 10.1146/annurev.mi.49.100195.003431 8561477

[B54] Covazzi Harriague A. Brino M. D. Zampini M. Albertelli G. Pruzzo C. Misic C. (2008). Vibrios in association with sedimentary crustaceans in three beaches of the northern Adriatic Sea (Italy). *Mar. Pollut. Bull.* 56 574–579 10.1016/j.marpolbul.2007.12.011 18243247

[B55] Coyne V. E. al-Harthi L. (1992). Induction of melanin biosynthesis in *Vibrio cholerae*. *Appl. Environ. Microbiol.* 58 2861–2865 1444398 10.1128/aem.58.9.2861-2865.1992 PMC183019

[B56] Dawson M. P. Humphrey B. A. Marshall K. C. (1981). Adhesion: a tactic in the survival strategy of a marine *Vibrio* during starvation. *Curr. Microbiol.* 6 195–199 10.1007/BF01566971

[B57] de Magny G. C. Mozumder P. K. Grim C. J. Hasan N. A. Naser M. N. Alam M. (2011). Role of zooplankton diversity in *Vibrio cholerae* population dynamics and in the incidence of cholera in the Bangladesh Sundarbans. *Appl. Environ. Microbiol.* 77 6125–6132 10.1128/AEM.01472-10 21764957 PMC3165371

[B58] Deriu A. Sechi L. A. Molicotti P. Spanu M. L. Zanetti S. (2002). Virulence genes in halophilic *Vibrio* spp. isolated in common mussels. *New Microbiol.* 25 93–96 11837398

[B59] Desmarchelier P. Wong F. Mallard K. (1995). An epidemiological study of *Vibrio cholerae* O1 in the Australian environment based on rRNA gene polymorphisms. *Epidemiol. Infect.* 115 435–446 10.1017/S0950268800058593 8557075 PMC2271589

[B60] Dickinson G. Lim K. Y. Jiang S. C. (2013). Quantitative microbial risk assessment of pathogenic *Vibrios* in marine recreational waters of Southern California. *Appl. Environ. Microbiol.* 79 294–302 10.1128/AEM.02674-12 23104412 PMC3536113

[B61] Eiler A. Gonzalez-Rey C. Allen S. Bertilsson S. (2007). Growth response of *Vibrio cholerae* and other *Vibrio* spp. to cyanobacterial dissolved organic matter and temperature in brackish water *FEMS Microbiol. Ecol.* 60 411–418 10.1111/j.1574-6941.2007.00303.x 17386033

[B62] Eiler A. Johansson M. Bertilsson S. (2006). Environmental influences on *Vibrio* populations in northern temperate and boreal coastal waters (Baltic and Skagerrak Seas). *Appl. Environ. Microbiol.* 72 6004–6011 10.1128/AEM.00917-06 16957222 PMC1563599

[B63] Epstein P. R. (1993). Algal blooms in the spread and persistence of cholera. *Biosystems* 31 209–221 10.1016/0303-2647(93)90050-M 8155853

[B64] Epstein S. (2009). “General model of microbial uncultivability,” in *Uncultivated Microorganisms* ed. Epstein S. S. (Berlin: Springer) 131–159

[B65] Erken M. Weitere M. Kjelleberg S. McDougald D. (2011). In situ grazing resistance of *Vibrio cholerae* in the marine environment. *FEMS Microbiol. Ecol.* 76 504–512 10.1111/j.1574-6941.2011.01067.x 21314704

[B66] Faruque S. M. Chowdhury N. Kamruzzaman M. Dziejman M. Rahman M. H. Sack D. A. (2004). Genetic diversity and virulence potential of environmental *Vibrio cholerae* population in a cholera-endemic area. *Proc. Natl. Acad. Sci. U.S.A.* 101 2123–2128 10.1073/pnas.0308485100 14766976 PMC357062

[B67] Faruque S. M. Islam M. J. Ahmad Q. S. Faruque A. S. G. Sack D. A. Nair G. B. (2005). Self-limiting nature of seasonal cholera epidemics: role of host-mediated amplification of phage. *Proc. Natl. Acad. Sci. U.S.A.* 102 6119–6124 10.1073/pnas.0502069102 15829587 PMC1087956

[B68] Focareta T. Manning P. A. (1991). Distinguishing between the extracellular DNases of *Vibrio cholerae* and development of a transformation system. *Mol. Microbiol.* 5 2547–2555 10.1111/j.1365-2958.1991.tb02101.x 1791765

[B69] Fong J. C. Karplus K. Schoolnik G. K. Yildiz F. H. (2006). Identification and characterization of RbmA, a novel protein required for the development of rugose colony morphology and biofilm structure in *Vibrio cholerae*. *J. Bacteriol.* 188 1049–1059 10.1128/JB.188.3.1049-1059.2006 16428409 PMC1347326

[B70] Fong J. C. Syed K. A. Klose K. E. Yildiz F. H. (2010). Role of *Vibrio* polysaccharide (vps) genes in VPS production, biofilm formation and *Vibrio cholerae* pathogenesis. *Microbiology* 156 2757–2769 10.1099/mic.0.040196-0 20466768 PMC3068689

[B71] Fong J. C. Yildiz F. H. (2007). The rbmBCDEF gene cluster modulates development of rugose colony morphology and biofilm formation in *Vibrio cholerae*. *J. Bacteriol.* 189 2319–2330 10.1128/JB.01569-06 17220218 PMC1899372

[B72] Franco A. A. Fix A. D. Prada A. Paredes E. Palomino J. C. Wright A. C. (1997). Cholera in Lima, Peru, correlates with prior isolation of *Vibrio cholerae* from the environment. *Am. J. Epidemiol.* 146 1067–1075 10.1093/oxfordjournals.aje.a009235 9420531

[B73] Freter R O’Brien P. C. (1981). Role of chemotaxis in the association of motile bacteria with intestinal mucosa: chemotactic responses of *Vibrio cholerae* and description of motile nonchemotactic mutants. *Infect. Immun.* 34 215–221 7298183 10.1128/iai.34.1.215-221.1981 PMC350845

[B74] Goldberg M. B. Boyko S. A. Calderwood S. B. (1991). Positive transcriptional regulation of an iron-regulated virulence gene in *Vibrio cholerae*. *Proc. Natl. Acad. Sci. U.S.A.* 88 1125–1129 10.1073/pnas.88.4.1125 1705025 PMC50969

[B75] González-Escalona N. Fey A. Höfle M. G. Espejo R. T. A. Guzmán C. (2006). Quantitative reverse transcription polymerase chain reaction analysis of *Vibrio cholerae* cells entering the viable but non-culturable state and starvation in response to cold shock. *Environ. Microbiol.* 8 658–666 10.1111/j.1462-2920.2005.00943.x 16584477

[B76] Gosink K. K. Kobayashi R. Kawagishi I. Hase C. C. (2002). Analyses of the roles of the three cheA homologs in chemotaxis of *Vibrio cholerae*. *J. Bacteriol.* 184 1767–1771 10.1128/JB.184.6.1767-1771.2002 11872729 PMC134905

[B77] Griffiths G. L. Sigel S. P. Payne S. M. Neilands J. B. (1984). Vibriobactin, a siderophore from *Vibrio cholerae*. *J. Biol. Chem.* 259 383–385 6706943

[B78] Grim C. J. Jaiani E. Whitehouse C. A. Janelidze N. Kokashvili T. Tediashvili M. (2010). Detection of toxigenic *Vibrio cholerae* O1 in freshwater lakes of the former Soviet Republic of Georgia. *Environ. Microbiol. Rep.* 2 2–6 10.1111/j.1758-2229.2009.00073.x 23765992

[B79] Gurbanov S. Akhmadov R. Shamkhalova G. Akhmadova S. Haley B. Colwell R. R. (2011). Occurrence of *Vibrio cholerae* in municipal and natural waters and incidence of cholera in Azerbaijan. *Ecohealth* 8 468–477 10.1007/s10393-012-0756-8 22451165

[B80] Hahn M. W Höfle M. G. (2001). Grazing of protozoa and its effect on populations of aquatic bacteria. *FEMS Microbiol. Ecol.* 35 113–121 10.1111/j.1574-6941.2001.tb00794.x 11295449

[B81] Haley B. J. Chen A. Grim C. J. Clark P. Diaz C. M. Taviani E. (2012). *Vibrio cholerae* in a historically cholera-free country. *Environ. Microbiol. Rep.* 4 381–389 10.1111/j.1758-2229.2012.00332.x 23185212 PMC3505037

[B82] Hall-Stoodley L. Costerton J. W. Stoodley P. (2004). Bacterial biofilms: from the natural environment to infectious diseases. *Nat. Rev. Microbiol.* 2 95–108 10.1038/nrmicro821 15040259

[B83] Halpern M. (2011). “Chironomids and *Vibrio cholerae*,” in *Beneficial Microorganisms in Multicellular Life Forms* eds Rosenberg E. Gophna U. (Berlin: Springer) 43–56

[B84] Halpern M. Broza Y. B. Mittler S. Arakawa E. Broza M. (2004). Chironomid egg masses as a natural reservoir of *Vibrio cholerae* non-O1 and non-O139 in freshwater habitats. *Microb. Ecol.* 47 341–349 10.1007/s00248-003-2007-6 14681736

[B85] Halpern M. Gancz H. Broza M. Kashi Y. (2003). *Vibrio cholerae* hemagglutinin/protease degrades chironomid egg masses. *Appl. Environ. Microbiol.* 69 4200–4204 10.1128/AEM.69.7.4200-4204.2003 12839800 PMC165141

[B86] Halpern M. Landsberg O. Raats D. Rosenberg E. (2007). Culturable and VBNC *Vibrio cholerae*: interactions with chironomid egg masses and their bacterial population. *Microb. Ecol.* 53 285–293 10.1007/s00248-006-9094-0 17186156

[B87] Halpern M. Raats D. Lavion R. Mittler S. (2006). Dependent population dynamics between chironomids (nonbiting midges) and *Vibrio cholerae*. *FEMS Microbiol. Ecol.* 55 98–104 10.1111/j.1574-6941.2005.00020.x 16420618

[B88] Halpern M. Senderovich Y. Izhaki I. (2008). Waterfowl: the missing link in epidemic and pandemic cholera dissemination? *PLoS Pathog*. 4:e1000173 10.1371/journal.ppat.1000173 PMC2565833 18974827

[B89] Hamana K. Matsuzaki S. (1982). Widespread occurrence of norspermidine and norspermine in eukaryotic algae. *J. Biochem.* 91 1321–1328 7096289 10.1093/oxfordjournals.jbchem.a133818

[B90] Hammer B. K. Bassler B. L. (2003). Quorum sensing controls biofilm formation in *Vibrio cholerae*. *Mol. Microbiol.* 50 101–104 10.1046/j.1365-2958.2003.03688.x 14507367

[B91] Haugo A. J. Watnick P. I. (2002). *Vibrio cholerae* CytR is a repressor of biofilm development. *Mol. Microbiol.* 45 471–483 10.1046/j.1365-2958.2002.03023.x 12123457 PMC2515492

[B92] Heidelberg J. F. Eisen J. A. Nelson W. C. Clayton R. A. Gwinn M. L. Dodson R. J. (2000). DNA sequence of both chromosomes of the cholera pathogen *Vibrio cholerae*. *Nature* 406 477–483 10.1038/35020000 10952301 PMC8288016

[B93] Hernroth B. Lothigius Å. Bölin I. (2010). Factors influencing survival of enterotoxigenic *Escherichia coli*, *Salmonella enterica* (serovar *Typhimurium*) and *Vibrio parahaemolyticus* in marine environments. *FEMS Microbiol. Ecol.* 71 272–280 10.1111/j.1574-6941.2009.00803.x 19930458

[B94] Hill V. R. Cohen N. Kahler A. M. Jones J. L. Bopp C. A. Marano N. (2011). Toxigenic *Vibrio cholerae* O1 in water and seafood, Haiti. *Emerg. Infect. Dis.* 17 2147 10.3201/eid1711.110748 PMC3310574 22099121

[B95] Hood M. A. Winter P. A. (1997). Attachment of *Vibrio cholerae* under various environmental conditions and to selected substrates. *FEMS Microbiol. Ecol.* 22 215–223 10.1111/j.1574-6941.1997.tb00373.x

[B96] Houot L. Chang S. Pickering B. S. Absalon C. Watnick P. I. (2010). The phosphoenolpyruvate phosphotransferase system regulates *Vibrio cholerae* biofilm formation through multiple independent pathways. *J. Bacteriol.* 192 3055–3067 10.1128/JB.00213-10 20400550 PMC2901703

[B97] Houot L. Watnick P. I. (2008). A novel role for enzyme I of the *Vibrio cholerae* phosphoenolpyruvate phosphotransferase system in regulation of growth in a biofilm. *J. Bacteriol.* 190 311–320 10.1128/JB.01410-07 17981973 PMC2223720

[B98] Hranitzky K. W. Mulholland A. Larson A. D. Eubanks E. R. Hart L. T. (1980). Characterization of a flagellar sheath protein of *Vibrio cholerae*. *Infect. Immun.* 27 597–603 7380541 10.1128/iai.27.2.597-603.1980 PMC550806

[B99] Hunt D. E. Gevers D. Vahora N. M. Polz M. F. (2008). Conservation of the chitin utilization pathway in the Vibrionaceae. *Appl. Environ. Microbiol.* 74 44–51 10.1128/AEM.01412-07 17933912 PMC2223224

[B100] Huq A. Sack R. B. Nizam A. Longini I. M. Nair G. B. Ali A. (2005). Critical factors influencing the occurrence of *Vibrio cholerae* in the environment of Bangladesh. *Appl. Environ. Microbiol.* 71 4645–4654 10.1128/AEM.71.8.4645-4654.2005 16085859 PMC1183289

[B101] Huq A. Small E. West P. Huq M. Rahman R. Colwell R. R. (1983). Ecological relationship between *Vibrio cholerae* and planktonic copepods. *Appl. Environ. Microbiol.* 45 275–283 6337551 10.1128/aem.45.1.275-283.1983 PMC242265

[B102] Huq A. Xu B. Chowdhury M. A. Islam M. S. Montilla R. Colwell R. R. (1996). A simple filtration method to remove plankton-associated *Vibrio cholerae* in raw water supplies in developing countries. *Appl. Environ. Microbiol.* 62 2508–2512 8779590 10.1128/aem.62.7.2508-2512.1996 PMC168033

[B103] Huq A. Yunus M. Sohel S. S. Bhuiya A. Emch M. Luby S. P. (2010). Simple sari cloth filtration of water is sustainable and continues to protect villagers from cholera in Matlab, Bangladesh. *MBio* 1 e0003410 10.1128/mBio.00034-10 PMC2912662 20689750

[B104] Ishikawa T. Rompikuntal P. K. Lindmark B. Milton D. L. Wai S. N. (2009). Quorum sensing regulation of the two hcp alleles in *Vibrio cholerae* O1 strains. *PLoS ONE* 4:e6734 10.1371/journal.pone.0006734 PMC2726435 19701456

[B105] Islam A. Labbate M. Djordjevic S. P. Alam M. Darling A. Melvold J. (2013). Indigenous *Vibrio cholerae* strains from a non-endemic region are pathogenic. *Open Biol.* 3 120181 10.1098/rsob.120181 PMC3603452 23407641

[B106] Islam M. Hasan M. Miah M. Yunus M. Zaman K. Albert M. (1994). Isolation of *Vibrio cholerae* O139 synonym Bengal from the aquatic environment in Bangladesh: implications for disease transmission. *Appl. Environ. Microbiol.* 60 1684–1686 8017948 10.1128/aem.60.5.1684-1686.1994 PMC201539

[B107] Islam M. S. Drasar B. S. Bradley D. J. (1989). Attachment of toxigenic *Vibrio cholerae* 01 to various freshwater plants and survival with a filamentous green alga, *Rhizoclonium fontanum*. *J. Trop. Med. Hyg.* 92 396–401 2607573

[B108] Islam M. S. Rahim Z. Alam M. J. Begum S. Moniruzzaman S. M. Umeda A. (1999). Association of *Vibrio cholerae* O1 with the cyanobacterium, *Anabaena* sp.,elucidated by polymerase chain reaction and transmission electron microscopy. *Trans. R. Soc. Trop. Med. Hyg.* 93 36–40 10.1016/S0035-9203(99)90171-2 10492786

[B109] Jahid I. K. Silva A. J. Benitez J. A. (2006). Polyphosphate stores enhance the ability of *Vibrio cholerae* to overcome environmental stresses in a low-phosphate environment. *Appl. Environ. Microbiol.* 72 7043–7049 10.1128/AEM.00924-06 16950899 PMC1636151

[B110] Jain V. Dongre M. Raychaudhuri S. (2006). Interaction of *Vibrio cholerae* O139 with an intestinal parasite, *Entamoeba histolytica*. *J. Med. Microbiol.* 55 1755–1756 10.1099/jmm.0.46812-0 17108284

[B111] Jiang S. C. (2001). *Vibrio cholerae* in recreational beach waters and tributaries of Southern California. *Hydrobiologia* 460 157–164 10.1023/A:1013152407425

[B112] Jobling M. G. Holmes R. K. (1997). Characterization of hapR, a positive regulator of the *Vibrio cholerae* HA/protease gene hap, and its identification as a functional homologue of the *Vibrio harveyi* luxR gene. *Mol. Microbiol.* 26 1023–1034 10.1046/j.1365-2958.1997.6402011.x 9426139

[B113] Johnson C. Flowers A. Noriea N. Zimmerman A. Bowers J. Depaola A. (2010). Relationships between environmental factors and pathogenic *Vibrios* in the Northern Gulf of Mexico. *Appl. Environ. Microbiol.* 76 7076–7084 10.1128/AEM.00697-10 20817802 PMC2976234

[B114] Johnson C. N. Bowers J. C. Griffitt K. J. Molina V. Clostio R. W. Pei S. (2012). Ecology of *Vibrio parahaemolyticus* and *Vibrio vulnificus* in the coastal and estuarine waters of Louisiana, Maryland, Mississippi, and Washington (United States). *Appl. Environ. Microbiol.* 78 7249–7257 10.1128/AEM.01296-12 22865080 PMC3457101

[B115] Jubair M. Morris J. G. Jr. Ali A. (2012). Survival of *Vibrio cholerae* in nutrient-poor environments is associated with a novel “persister” phenotype. *PLoS ONE* 7:e45187 10.1371/journal.pone.0045187 PMC3445476 23028836

[B116] Jutla A. S. Akanda A. S. Griffiths J. K. Colwell R. R. Islam S. (2011). Warming oceans, phytoplankton, and river discharge: implications for cholera outbreaks. *Am. J. Trop. Med. Hyg.* 85 303–308 10.4269/ajtmh.2011.11-0181 21813852 PMC3144830

[B117] Kamruzzaman M. Udden S. N. Cameron D. E. Calderwood S. B. Nair G. B. Mekalanos J. J. (2010). Quorum-regulated biofilms enhance the development of conditionally viable, environmental *Vibrio cholerae*. *Proc. Natl. Acad. Sci. U.S.A.* 107 1588–1593 10.1073/pnas.0913404107 20080633 PMC2824409

[B118] Kan B. Habibi H. Schmid M. Liang W. Wang R. Wang D. (2004). Proteome comparison of *Vibrio cholerae* cultured in aerobic and anaerobic conditions. *Proteomics* 4 3061–3067 10.1002/pmic.200400944 15378743

[B119] Karatan E. Duncan T. R. Watnick P. I. (2005). NspS, a predicted polyamine sensor, mediates activation of *Vibrio cholerae* biofilm formation by norspermidine. *J. Bacteriol.* 187 7434–7443 10.1128/JB.187.21.7434-7443.2005 16237027 PMC1273002

[B120] Kierek K. Watnick P. I. (2003). Environmental determinants of *Vibrio cholerae* biofilm development. *Appl. Environ. Microbiol.* 69 5079–5088 10.1128/AEM.69.9.5079-5088.2003 12957889 PMC194957

[B121] Kiiyukia C. Nakajima A. Nakai T. Muroga K. Kawakami H. Hashimoto H. (1992). *Vibrio cholerae* non-O1 isolated from ayu fish (*Plecoglossus altivelis*) in Japan. *Appl. Environ. Microbiol.* 58 3078–3082 1280062 10.1128/aem.58.9.3078-3082.1992 PMC183051

[B122] Kirn T. J. Jude B. A. Taylor R. K. (2005). A colonization factor links *Vibrio cholerae* environmental survival and human infection. *Nature* 438 863–866 10.1038/nature04249 16341015

[B123] Kirs M. Depaola A. Fyfe R. Jones J. Krantz J. Van Laanen A. (2011). A survey of oysters *Crassostrea gigas* in New Zealand for *Vibrio parahaemolyticus* and *Vibrio vulnificus*. *Int. J. Food Microbiol.* 147 149–153 10.1016/j.ijfoodmicro.2011.03.012 21501884

[B124] Kirschner A. K. T. Schlesinger J. Farnleitner A. H. Hornek R. Süß B. Golda B. (2008). Rapid growth of planktonic *Vibrio cholerae* non-O1/non-O139 strains in a large alkaline lake in Austria: dependence on temperature and dissolved organic carbon quality. *Appl. Environ. Microbiol.* 74 2004–2015 10.1128/AEM.01739-07 18245230 PMC2292586

[B125] Kojima S. Yamamoto K. Kawagishi I. Homma M. (1999). The polar flagellar motor of *Vibrio cholerae* is driven by an Na^+^ motive force. *J. Bacteriol.* 181 1927–1930 10074090 10.1128/jb.181.6.1927-1930.1999 PMC93596

[B126] Kong I. S. Bates T. C. Hülsmann A. Hassan H. Smith B. E. Oliver J. D. (2004). Role of catalase and oxyR in the viable but nonculturable state of *Vibrio vulnificus*. *FEMS Microbiol. Ecol.* 50 133–142 10.1016/j.femsec.2004.06.004 19712354

[B127] Krasteva P. V. Fong J. C. Shikuma N. J. Beyhan S. Navarro M. V. Yildiz F. H. (2010). *Vibrio cholerae* VpsT regulates matrix production and motility by directly sensing cyclic di-GMP. *Science* 327 866–868 10.1126/science.1181185 20150502 PMC2828054

[B128] Lama J. R. Seas C. R. León-Barúa R. Gotuzzo E. Sack R. B. (2011). Environmental temperature, cholera, and acute diarrhoea in adults in Lima, Peru. *J. Health Popul. Nutr.* 22 399–403 15663172

[B129] Leal N. C. De Araújo Figueiroa Â. C. T. Cavalcanti V. O. Da Silva S. C. Leal-Balbino T. C. De Almeida A. M. P. (2008). Characterization of *Vibrio cholerae* isolated from the aquatic basins of the State of Pernambuco, Brazil. *Trans. R. Soc. Trop. Med. Hyg.* 102 272–276 10.1016/j.trstmh.2007.12.008 18258274

[B130] Lipp E. K. Rivera I. N. Gil A. I. Espeland E. M. Choopun N. Louis V. R. (2003). Direct detection of *Vibrio cholerae* and ctxA in Peruvian coastal water and plankton by PCR. *Appl. Environ. Microbiol.* 69 3676–3680 10.1128/AEM.69.6.3676-3680.2003 12788781 PMC161524

[B131] Liu Z. Stirling F. R. Zhu J. (2007). Temporal quorum-sensing induction regulates *Vibrio cholerae* biofilm architecture. *Infect. Immun.* 75 122–126 10.1128/IAI.01190-06 17074850 PMC1828391

[B132] Lizárraga-Partida M. L. Mendez-Gomez E. Rivas-Montano A. M. Vargas-Hernandez E. Portillo-Lopez A. Gonzalez-Ramirez A. R. (2009). Association of *Vibrio cholerae* with plankton in coastal areas of Mexico. *Environ. Microbiol.* 11 201–208 10.1111/j.1462-2920.2008.01753.x 18793311

[B133] Lo Scrudato M. Blokesch M. (2012). The regulatory network of natural competence and transformation of *Vibrio cholerae*. *PLoS Genet.* 8:e1002778 10.1371/journal.pgen.1002778 PMC3380833 22737089

[B134] Lobitz B. Beck L. Huq A. Wood B. Fuchs G. Faruque A. S. (2000). Climate and infectious disease: use of remote sensing for detection of *Vibrio cholerae* by indirect measurement. *Proc. Natl. Acad. Sci. U.S.A.* 97 1438–1443 10.1073/pnas.97.4.1438 10677480 PMC26452

[B135] Lock R. Öhman L. Dahlgren C. (1987). Phagocytic recognition mechanisms in human granulocytes and *Acanthamoeba castellanii* using type 1 fimbriated *Escherichia coli* as phagocytic prey. *FEMS Microbiol. Lett.* 44 135–140

[B136] Louis V. R. Russek-Cohen E. Choopun N. Rivera I. N. G. Gangle B. Jiang S. C. (2003). Predictability of *Vibrio cholerae* in Chesapeake Bay. *Appl. Environ. Microbiol.* 69 2773–2785 10.1128/AEM.69.5.2773-2785.2003 12732548 PMC154498

[B137] Macek M. Carlos G. Memije P Ramãrez P. (1997). Ciliate–*Vibrio cholerae* interactions within a microbial loop: an experimental study. *Aquat. Microb. Ecol.* 13 257–266 10.3354/ame013257

[B138] MacIntyre D. L. Miyata S. T. Kitaoka M. Pukatzki S. (2010). The *Vibrio cholerae* type VI secretion system displays antimicrobial properties. *Proc. Natl. Acad. Sci. U.S.A.* 107 19520–19524 10.1073/pnas.1012931107 20974937 PMC2984155

[B139] Maina A. N. Mwaura F. B. Oyugi J. Goulding D. Toribio A. L. Kariuki S. (2013). Characterization of *Vibrio cholerae* bacteriophages isolated from the environmental waters of the Lake Victoria region of Kenya. *Curr. Microbiol.* 1–7 [Epub ahead of print] 10.1007/s00284-013-0447-x PMC4173113 23982202

[B140] Martin J. H. (1992). “Iron as a limiting factor in oceanic productivity,” in *Primary Productivity and Biogeochemical Cycles in the Sea* eds Falkowski P. Woodhead A. (New York: Plenum Press) 123–137

[B141] Martin J. H Michael Gordon R. (1988). Northeast Pacific iron distributions in relation to phytoplankton productivity. *Deep-Sea Res.* 35 177–196 10.1016/0198-0149(88)90035-0

[B142] Martinelli Filho J. E. Lopes R. M. Rivera I. N. G. Colwell R. R. (2010). *Vibrio cholerae* O1 detection in estuarine and coastal zooplankton. *J. Plankton Res.* 33 51–62 10.1093/plankt/fbq093

[B143] Martïnez J. P. Falomir M. P. Gozalbo D. (2009). “Chitin: a structural biopolysaccharide,” in *eLS*. John Wiley & Sons, Ltd.

[B144] Martïnez Pérez M. E. Macek M. Castro Galván M. T. (2004). Do protozoa control the elimination of *Vibrio cholerae* in brackish water? *Int. Rev. Hydrobiol.* 89 215–227 10.1002/iroh.200310644

[B145] Matz C. Kjelleberg S. (2005). Off the hook – how bacteria survive protozoan grazing. *Trends Microbiol.* 13 302–307 10.1016/j.tim.2005.05.009 15935676

[B146] Matz C. McDougald D. Moreno A. M. Yung P. Y. Yildiz F. H. Kjelleberg S. (2005). Biofilm formation and phenotypic variation enhance predation-driven persistence of *Vibrio cholerae*. *Proc. Natl. Acad. Sci. U.S.A.* 102 16819–16824 10.1073/pnas.0505350102 16267135 PMC1283802

[B147] Maugeri T. Caccamo D. Gugliandolo C. (2001). Potentially pathogenic Vibrios in brackish waters and mussels. *J. Appl. Microbiol.* 89 261–266 10.1046/j.1365-2672.2000.01096.x 10971757

[B148] McDougald D. Rice S. A. Kjelleberg S. (1999). New perspectives on the viable but nonculturable response. *Biologia-bratislava* 54 617–624

[B149] McDougald D. Rice S. A. Weichart D. Kjelleberg S. (1998). Nonculturability: adaptation or debilitation? *FEMS Microbiol. Ecol.* 25 1–9 10.1111/j.1574-6941.1998.tb00455.x

[B150] Meibom K. L. Li X. B. Nielsen A. T. Wu C. Y. Roseman S. Schoolnik G. K. (2004). The *Vibrio cholerae* chitin utilization program. *Proc. Natl. Acad. Sci. U.S.A.* 101 2524–2529 10.1073/pnas.0308707101 14983042 PMC356983

[B151] Merrell D. S. Camilli A. (1999). The cadA gene of *Vibrio cholerae* is induced during infection and plays a role in acid tolerance. *Mol. Microbiol.* 34 836–849 10.1046/j.1365-2958.1999.01650.x 10564522

[B152] Miller V. L. Mekalanos J. J. (1988). A novel suicide vector and its use in construction of insertion mutations: osmoregulation of outer membrane proteins and virulence determinants in *Vibrio cholerae* requires toxR. *J. Bacteriol.* 170 2575–2583 2836362 10.1128/jb.170.6.2575-2583.1988 PMC211174

[B153] Mishra A. Taneja N. Sharma M. (2012). Viability kinetics, induction, resuscitation and quantitative real-time polymerase chain reaction analyses of viable but nonculturable *Vibrio cholerae* O1 in freshwater microcosm. *J. Appl. Microbiol.* 112 945–953 10.1111/j.1365-2672.2012.05255.x 22324483

[B154] Mitta G. Vandenbulcke F. Roch P. (2000). Original involvement of antimicrobial peptides in mussel innate immunity. *FEBS Lett.* 486 185–190 10.1016/S0014-5793(00)02192-X 11119700

[B155] Miyagi K. Nakano T. Yagi T. Hanafusa M. Imura S. Honda T. (2003). Survey of *Vibrio cholerae* O1 and its survival over the winter in marine water of Port of Osaka. *Epidemiol. Infect.* 131 613–619 10.1017/S0950268803008756 12948359 PMC2870000

[B156] Miyata S. T. Kitaoka M. Brooks T. M. McAuley S. B. Pukatzki S. (2011). *Vibrio cholerae* requires the type VI secretion system virulence factor VasX to kill *Dictyostelium discoideum*. *Infect. Immun.* 79 2941–2949 10.1128/IAI.01266-10 21555399 PMC3191968

[B157] Miyata S. T. Kitaoka M. Wieteska L. Frech C. Chen N. Pukatzki S. (2010). The *Vibrio cholerae* type VI secretion system: evaluating its role in the human disease cholera. *Front. Microbiol.* 1:117 10.3389/fmicb.2010.00117 PMC3095397 21607085

[B158] Mizunoe Y. Wai S. N. Takade A. Yoshida S. I. (1999). Restoration of culturability of starvation-stressed and low-temperature-stressed *Escherichia coli* O157 cells by using H_2_O_2_-degrading compounds. *Arch. Microbiol.* 172 63–67 10.1007/s002030050741 10398754

[B159] Moorthy S. Watnick P. I. (2004). Genetic evidence that the *Vibrio cholerae* monolayer is a distinct stage in biofilm development. *Mol. Microbiol.* 52 573–587 10.1111/j.1365-2958.2004.04000.x 15066042 PMC2501105

[B160] Morris J. G. Jr. Sztein M. B. Rice E. W. Nataro J. P. Losonsky G. A. Panigrahi P. (1996). *Vibrio cholerae* O1 can assume a chlorine-resistant rugose survival form that is virulent for humans. *J. Infect. Dis.* 174 1364–1368 10.1093/infdis/174.6.1364 8940236

[B161] Mouriño-Pérez R. R. Worden A. Z. Azam F. (2003). Growth of *Vibrio cholerae* O1 in red tide waters off California. *Appl. Environ. Microbiol.* 69 6923–6931 10.1128/AEM.69.11.6923-6931.2003 14602656 PMC262290

[B162] Muller J. Miller M. C. Nielsen A. T. Schoolnik G. K. Spormann A. M. (2007). vpsA- and luxO-independent biofilms of *Vibrio cholerae*. *FEMS Microbiol. Lett.* 275 199–206 10.1111/j.1574-6968.2007.00884.x 17697110

[B163] Murphree R. L. Tamplin M. L. (1995). Uptake and retention of *Vibrio cholerae* O1 in the Eastern oyster, *Crassostrea virginica*. *Appl. Environ. Microbiol.* 61 3656–3660 7487003 10.1128/aem.61.10.3656-3660.1995 PMC167666

[B164] Nalin D. Daya V. Reid A. Levine M. Cisneros L. (1979). Adsorption and growth of *Vibrio cholerae* on chitin. *Infect. Immun.* 25 768–770 489131 10.1128/iai.25.2.768-770.1979 PMC414510

[B165] Nesper J. Lauriano C. M. Klose K. E. Kapfhammer D. Kraiss A. Reidl J. (2001). Characterization of *Vibrio cholerae* O1 El Tor galU and galE mutants: influence on lipopolysaccharide structure, colonization, and biofilm formation. *Infect. Immun.* 69 435–445 10.1128/IAI.69.1.435-445.2001 11119535 PMC97901

[B166] Nilsson L. Oliver J. Kjelleberg S. (1991). Resuscitation of *Vibrio vulnificus* from the viable but nonculturable state. *J. Bacteriol.* 173 5054–5059 1860818 10.1128/jb.173.16.5054-5059.1991 PMC208195

[B167] Ogawa N. Tzeng C. M. Fraley C. D. Kornberg A. (2000). Inorganic polyphosphate in *Vibrio cholerae*: genetic, biochemical, and physiologic features. *J. Bacteriol.* 182 6687–6693 10.1128/JB.182.23.6687-6693.2000 11073913 PMC111411

[B168] Ogg J. E. Ryder R. A. Smith H. L. Jr (1989). Isolation of *Vibrio cholerae* from aquatic birds in Colorado and Utah. *Appl. Environ. Microbiol.* 55 95–99 2705773 10.1128/aem.55.1.95-99.1989 PMC184060

[B169] Olafsen J. A. Mikkelsen H. V. Giæver H. M Høvik Hansen G. (1993). Indigenous bacteria in hemolymph and tissues of marine bivalves at low temperatures. *Appl. Environ. Microbiol.* 59 1848–1854 16348962 10.1128/aem.59.6.1848-1854.1993 PMC182171

[B170] Oliver J. D. (2005). The viable but nonculturable state in bacteria. *J. Microbiol.* 43 93–100 15765062

[B171] Oliver J. D. (2010). Recent findings on the viable but nonculturable state in pathogenic bacteria. *FEMS Microbiol. Rev.* 34 415–425 20059548 10.1111/j.1574-6976.2009.00200.x

[B172] Oliver J. D. Hite F. McDougald D. Andon N. L. Simpson L. M. (1995). Entry into, and resuscitation from, the viable but nonculturable state by *Vibrio vulnificus* in an estuarine environment. *Appl. Environ. Microbiol.* 61 2624–2630 7618874 10.1128/aem.61.7.2624-2630.1995 PMC167534

[B173] Pearson G. D. Woods A. Chiang S. L. Mekalanos J. J. (1993). CTX genetic element encodes a site-specific recombination system and an intestinal colonization factor. *Proc. Natl. Acad. Sci. U.S.A.* 90 3750–3754 10.1073/pnas.90.8.3750 8475125 PMC46379

[B174] Pollitzer R. (1954). History of the disease. Cholera. *Bull. World Health Organ.* 10 421 PMC2542143 13160764

[B175] Pratt J. T. McDonough E. Camilli A. (2009). PhoB regulates motility, biofilms, and cyclic di-GMP in *Vibrio cholerae*. *J. Bacteriol.* 191 6632–6642 10.1128/JB.00708-09 19734314 PMC2795287

[B176] Preiss J. Romeo T. (1994). Molecular biology and regulatory aspects of glycogen biosynthesis in bacteria. *Prog. Nucleic Acid Res. Mol. Biol.* 47 299–329 10.1016/S0079-6603(08)60255-X 8016324

[B177] Pruzzo C. Gallo G. Canesi L. (2005). Persistence of Vibrios in marine bivalves: the role of interactions with haemolymph components. *Environ. Microbiol.* 7 761–772 10.1111/j.1462-2920.2005.00792.x 15892695

[B178] Pruzzo C. Tarsi R. Del Mar Lleò M. Signoretto C. Zampini M. Pane L. (2003). Persistence of adhesive properties in *Vibrio cholerae* after long-term exposure to sea water. *Environ. Microbiol.* 5 850–858 10.1046/j.1462-2920.2003.00498.x 14510838

[B179] Pruzzo C. Vezzulli L. Colwell R. R. (2008). Global impact of *Vibrio cholerae* interactions with chitin. *Environ. Microbiol.* 10 1400–1410 10.1111/j.1462-2920.2007.01559.x 18312392

[B180] Pukatzki S. Ma A. T. Revel A. T. Sturtevant D. Mekalanos J. J. (2007). Type VI secretion system translocates a phage tail spike-like protein into target cells where it cross-links actin. *Proc. Natl. Acad. Sci. U.S.A.* 104 15508–15513 10.1073/pnas.0706532104 17873062 PMC2000545

[B181] Pukatzki S. Ma A. T. Sturtevant D. Krastins B. Sarracino D. Nelson W. C. (2006). Identification of a conserved bacterial protein secretion system in *Vibrio cholerae* using the *Dictyostelium* host model system. *Proc. Natl. Acad. Sci. U.S.A.* 103 1528–1533 10.1073/pnas.0510322103 16432199 PMC1345711

[B182] Rashid A. Haley B. J. Rajabov M. Ahmadova S. Gurbanov S. Colwell R. R. (2013). Detection of *Vibrio cholerae* in environmental waters including drinking water reservoirs of Azerbaijan. *Environ. Microbiol. Rep.* 5 30–38 10.1111/j.1758-2229.2012.00369.x 23757128

[B183] Ravel J. Knight I. T. Monahan C. E. Hill R. T. Colwell R. R. (1995). Temperature-induced recovery of *Vibrio cholerae* from the viable but nonculturable state: growth or resuscitation? *Microbiology* 141 377–383 10.1099/13500872-141-2-377 7704268

[B184] Rawlings T. K. Ruiz G. M. Colwell R. R. (2007). Association of *Vibrio cholerae* O1 El Tor and O139 Bengal with the Copepods *Acartia tonsa* and *Eurytemora affinis*. *Appl. Environ. Microbiol.* 73 7926–7933 10.1128/AEM.01238-07 17951440 PMC2168156

[B185] Reguera G. Kolter R. (2005). Virulence and the environment: a novel role for *Vibrio cholerae* toxin-coregulated pili in biofilm formation on chitin. *J. Bacteriol.* 187 3551–3555 10.1128/JB.187.10.3551-3555.2005 15866944 PMC1112007

[B186] Rice E. W. Johnson C. J. Clark R. M. Fox K. R. Reasoner D. J. Dunnigan M. E. (1992). Chlorine and survival of “rugose” *Vibrio cholerae*. *Lancet* 340 740 10.1016/0140-6736(92)92289-R 1355849

[B187] Rinaudo M. (2006). Chitin and chitosan: properties and applications. *Prog. Polym. Sci.* 31 603–632 10.1016/j.progpolymsci.2006.06.001

[B188] Rowbotham T. J. (1980). Preliminary report on the pathogenicity of *Legionella pneumophila* for freshwater and soil amoebae. *J. Clin. Pathol.* 33 1179–1183 10.1136/jcp.33.12.1179 7451664 PMC1146371

[B189] Sá L. L. C. Vale E. R. V. Garza D. R Vicente A. C. P. (2012). *Vibrio cholerae* O1 from superficial water of the Tucunduba Stream, Brazilian Amazon. *Braz. J. Microbiol.* 43 635–638 10.1590/S1517-83822012000200027 24031874 PMC3768806

[B190] Sack D. A. Sack R. B. Nair G. B. Siddique A. K. (2004). Cholera. *Lancet* 363 223–233 10.1016/S0140-6736(03)15328-7 14738797

[B191] Sandström G. Saeed A. Abd H. (2010). *Acanthamoeba polyphaga* is a possible host for *Vibrio cholerae* in aquatic environments. *Exp. Parasitol.* 126 65–68 10.1016/j.exppara.2009.09.021 19815016

[B192] Schuster B. M. Tyzik A. L. Donner R. A. Striplin M. J. Almagro-Moreno S. Jones S. H. (2011). Ecology and genetic structure of a northern temperate *Vibrio cholerae* population related to toxigenic isolates. *Appl. Environ. Microbiol.* 77 7568–7575 10.1128/AEM.00378-11 21926213 PMC3209147

[B193] Seed K. D. Bodi K. L. Kropinski A. M. Ackermann H. W. Calderwood S. B. Qadri F. (2011). Evidence of a dominant lineage of *Vibrio cholerae*-specific lytic bacteriophages shed by cholera patients over a 10-year period in Dhaka, Bangladesh. *MBio* 2 10.1128/mBio.00334-10 PMC3037004 21304168

[B194] Seeligmann C. T. Mirande V. Tracanna B. C. Silva C. Aulet O. Cecilia M. (2008). Phytoplankton-linked viable non-culturable *Vibrio cholerae* O1 (VNC) from rivers in Tucumán, Argentina. *J. Plankton Res.* 30 367–377 10.1093/plankt/fbn008

[B195] Sen A. Ghosh A. N. (2005). New *Vibrio cholerae* O1 biotype El Tor bacteriophages. *Virol. J.* 2 28 10.1186/1743-422X-2-28 PMC1087507 15823200

[B196] Senderovich Y. Izhaki I. Halpern M. (2010). Fish as reservoirs and vectors of *Vibrio cholerae*. *PLoS ONE* 5:e8607 10.1371/journal.pone.0008607 PMC2797615 20066040

[B197] Senoh M. Ghosh-Banerjee J. Ramamurthy T. Hamabata T. Kurakawa T. Takeda M. (2010). Conversion of viable but nonculturable *Vibrio cholerae* to the culturable state by co-culture with eukaryotic cells. *Microbiol. Immunol.* 54 502–507 10.1111/j.1348-0421.2010.00245.x 20840148

[B198] Seper A. Fengler V. H. I. Roier S. Wolinski H. Kohlwein S. D. Bishop A. L. (2011). Extracellular nucleases and extracellular DNA play important roles in *Vibrio cholerae* biofilm formation. *Mol. Microbiol.* 82 1015–1037 10.1111/j.1365-2958.2011.07867.x 22032623 PMC3212620

[B199] Shikuma N. J. Hadfield M. G. (2010). Marine biofilms on submerged surfaces are a reservoir for *Escherichia coli* and *Vibrio cholerae*. *Biofouling* 26 39–46 10.1080/08927010903282814 20390555

[B200] Singh R. Narayan V. McLenachan P. Winkworth R. C. Mitra S. Lockhart P. J. (2012). Detection and diversity of pathogenic *Vibrio* from Fiji. *Environ. Microbiol. Rep.* 4 403–411 10.1111/j.1758-2229.2012.00344.x 23760825

[B201] Singleton F. L. Attwell R. W. Jangi M. S. Colwell R. R. (1982). Influence of salinity and organic nutrient concentration on survival and growth of *Vibrio cholerae* in aquatic microcosms. *Appl. Environ. Microbiol.* 43 1080–1085 6896621 10.1128/aem.43.5.1080-1085.1982 PMC244189

[B202] Snow J. (1855). *On the Mode of Communication of Cholera*. New Burlington Street, London: John Churchill

[B203] Srivastava D. Harris R. C. Waters C. M. (2011). Integration of cyclic di-GMP and quorum sensing in the control of vpsT and aphA in *Vibrio cholerae*. *J. Bacteriol.* 193 6331–6341 10.1128/JB.05167-11 21926235 PMC3209240

[B204] Srivastava D. Waters C. M. (2012). A tangled web: regulatory connections between quorum sensing and cyclic di-GMP. *J. Bacteriol.* 194 4485–4493 10.1128/JB.00379-12 22661686 PMC3415487

[B205] Stauder M. Vezzulli L. Pezzati E. Repetto B. Pruzzo C. (2010). Temperature affects *Vibrio cholerae* O1 El Tor persistence in the aquatic environment via an enhanced expression of GbpA and MSHA adhesins. *Environ. Microbiol. Rep.* 2 140–144 10.1111/j.1758-2229.2009.00121.x 23766009

[B206] Stocker R. Seymour J. R. (2012). Ecology and physics of bacterial chemotaxis in the ocean. *Microbiol. Mol. Biol. Rev.* 76 792–812 10.1128/MMBR.00029-12 23204367 PMC3510523

[B207] Sultan S. Z. Silva A. J. Benitez J. A. (2010). The PhoB regulatory system modulates biofilm formation and stress response in El Tor biotype *Vibrio cholerae*. *FEMS Microbiol. Lett.* 302 22–31 10.1111/j.1574-6968.2009.01837.x 19909344 PMC2792938

[B208] Sun S. Kjelleberg S. McDougald D. (2013). Relative contributions of *Vibrio* polysaccharide and quorum sensing to the resistance of *Vibrio cholerae* to predation by heterotrophic protists. *PLoS ONE* 8:e56338 10.1371/journal.pone.0056338 PMC3575383 23441178

[B209] Tall A. Hervio-Heath D. Teillon A. Boisset-Helbert C. Delesmont R. Bodilis J. (2013). Diversity of *Vibrio* spp. isolated at ambient environmental temperature in the eastern English Channel as determined by pyrH sequencing. *J. Appl. Microbiol.* 114 1713–1724 10.1111/jam.12181 23473469

[B210] Talledo M. Rivera I. N. Lipp E. K. Neale A. Karaolis D. Huq A. (2003). Characterization of a *Vibrio cholerae* phage isolated from the coastal water of Peru. *Environ. Microbiol.* 5 350–354 10.1046/j.1462-2920.2003.00411.x 12713461

[B211] Tamplin M. Gauzens A. Huq A. Sack D. Colwell R. R. (1990). Attachment of *Vibrio cholerae* serogroup O1 to zooplankton and phytoplankton of Bangladesh waters. *Appl. Environ. Microbiol.* 56 1977–1980 2383016 10.1128/aem.56.6.1977-1980.1990 PMC184543

[B212] Taviani E. Ceccarelli D. Lazaro N. Bani S. Cappuccinelli P. Colwell R. R. (2008). Environmental *Vibrio* spp., isolated in Mozambique, contain a polymorphic group of integrative conjugative elements and class 1 integrons. *FEMS Microbiol. Ecol.* 64 45–54 10.1111/j.1574-6941.2008.00455.x 18318712

[B213] Thom S. Warhurst D. Drasar B. S. (1992). Association of *Vibrio cholerae* with fresh water amoebae. *J. Med. Microbiol.* 36 303–306 10.1099/00222615-36-5-303 1588578

[B214] Thomas K. U. Joseph N. Raveendran O. Nair S. (2006). Salinity-induced survival strategy of *Vibrio cholerae* associated with copepods in Cochin backwaters. *Mar. Pollut. Bull.* 52 1425–1430 10.1016/j.marpolbul.2006.04.011 16764894

[B215] Turner J. W. Good B. Cole D. Lipp E. K. (2009). Plankton composition and environmental factors contribute to *Vibrio* seasonality. *ISME J.* 3 1082–1092 10.1038/ismej.2009.50 19421235

[B216] Turner J. W. Malayil L. Guadagnoli D. Cole D. Lipp E. K. (2013). Detection of *Vibrio parahaemolyticus, Vibrio vulnificus* and *Vibrio cholerae* with respect to seasonal fluctuations in temperature and plankton abundance. *Environ. Microbiol*. 10.1111/1462-2920.12246 [Epub ahead of print] 24024909

[B217] Unterweger D. Kitaoka M. Miyata S. T. Bachmann V. Brooks T. M. Moloney J. (2012). Constitutive type VI secretion system expression gives *Vibrio cholerae* intra- and interspecific competitive advantages. *PLoS ONE* 7:e48320 10.1371/journal.pone.0048320 PMC3482179 23110230

[B218] Vaitkevicius K. Lindmark B. Ou G. Song T. Toma C. Iwanaga M. (2006). A *Vibrio cholerae* protease needed for killing of *Caenorhabditis elegans* has a role in protection from natural predator grazing. *Proc. Natl. Acad. Sci. U.S.A.* 103 9280–9285 10.1073/pnas.0601754103 16754867 PMC1482601

[B219] Valeru S. Wai S. Saeed A. Sandström G. Abd H. (2012). ToxR of *Vibrio cholerae* affects biofilm, rugosity and survival with *Acanthamoeba castellanii*. *BMC Res. Notes* 5:33 10.1186/1756-0500-5-33 PMC3292481 22248371

[B220] Valeru S. P. Rompikuntal P. K. Ishikawa T. Vaitkevicius K. Sjoling A. Dolganov N. (2009). Role of melanin pigment in expression of *Vibrio cholerae* virulence factors. *Infect. Immun.* 77 935–942 10.1128/IAI.00929-08 19103773 PMC2643646

[B221] Vezzulli L. Brettar I. Pezzati E. Reid P. C. Colwell R. R. Höfle M. G. (2011). Long-term effects of ocean warming on the prokaryotic community: evidence from the vibrios. *ISME J.* 6 21–30 10.1038/ismej.2011.89 21753799 PMC3246245

[B222] Vezzulli L. Pezzati E. Moreno M. Fabiano M. Pane L. Pruzzo C. (2009). Benthic ecology of *Vibrio* spp. and pathogenic *Vibrio* species in a coastal Mediterranean environment (La Spezia Gulf, Italy). *Microb. Ecol.* 58 808–818 10.1007/s00248-009-9542-8 19543938

[B223] Vezzulli L. Pruzzo C. Huq A. Colwell R. R. (2010). Environmental reservoirs of *Vibrio cholerae* and their role in cholera. *Environ. Microbiol. Rep.* 2 27–33 10.1111/j.1758-2229.2009.00128.x 23765995

[B224] Vimala B. Thong K. L. Chong V. (2010). Isolation, detection and genomic differentiation of *Vibrio cholerae* and *Vibrio parahaemolyticus* in Bachok, Kelantan. *Malays. J. Sci.* 29 1–10

[B225] Vugia D. J. Shefer A. M. Douglas J. Greene K. D. Bryant R. G. Werner S. B. (1997). Cholera from raw seaweed transported from the Philippines to California. *J. Clin. Microbiol.* 35 284–285 8968927 10.1128/jcm.35.1.284-285.1997 PMC229558

[B226] Wackett L. Orme-Johnson W. Walsh C. (1989). “Transition metal enzymes in bacterial metabolism,” in *Metal Ions and Bacteria* eds Beveridge T. J. Doyle R. J. (New York, NY: Wiley-Interscience) 165–206

[B227] Wai S. N. Mizunoe Y. Takade A. Kawabata S. I. Yoshida S. I. (1998). *Vibrio cholerae* O1 Strain TSI-4 produces the exopolysaccharide materials that determine colony morphology, stress resistance, and biofilm formation. *Appl. Environ. Microbiol.* 64 3648–3655 9758780 10.1128/aem.64.10.3648-3655.1998 PMC106490

[B228] Waldor M. K. Mekalanos J. J. (1996). Lysogenic conversion by a filamentous phage encoding cholera toxin. *Science* 272 1910–1914 10.1126/science.272.5270.1910 8658163

[B229] Watnick P. I. Kolter R. (1999). Steps in the development of a *Vibrio cholerae* El Tor biofilm. *Mol. Microbiol.* 34 586–595 10.1046/j.1365-2958.1999.01624.x 10564499 PMC2860543

[B230] Watnick P. I. Lauriano C. M. Klose K. E. Croal L. Kolter R. (2001). The absence of a flagellum leads to altered colony morphology, biofilm development and virulence in *Vibrio cholerae* O139. *Mol. Microbiol.* 39 223–235 10.1046/j.1365-2958.2001.02195.x 11136445 PMC2860545

[B231] Wei Y. Kirby A. Levin B. R. (2011). The population and evolutionary dynamics of *Vibrio cholerae* and its bacteriophage: conditions for maintaining phage-limited communities. *Am. Nat.* 178 715–725 10.1086/662677 22089867

[B232] Weichart D. McDougald D. Jacobs D. Kjelleberg S. (1997). In situ analysis of nucleic acids in cold-induced nonculturable *Vibrio vulnificus*. *Appl. Environ. Microbiol*. 63 2754–2758 9212422 10.1128/aem.63.7.2754-2758.1997 PMC168571

[B233] Winiecka-Krusnell J. Linder E. (2001). Bacterial infections of free-living amoebae. *Res. Microbiol.* 152 613–619 10.1016/S0923-2508(01)01240-2 11605981

[B234] Worden A. Z. Seidel M. Smriga S. Wick A. Malfatti F. Bartlett D. (2006). Trophic regulation of *Vibrio cholerae* in coastal marine waters. *Environ. Microbiol.* 8 21–29 10.1111/j.1462-2920.2005.00863.x 16343318

[B235] Wright A. C. Harwood V. J. (2013). “Vibrios,” in *Foodborne Infections and Intoxications* 4th Edn eds Glenn J. M. Morris P. (San Diego: Academic Press) 113–128

[B236] Wyckoff E. E. Mey A. R. Leimbach A. Fisher C. F. Payne S. M. (2006). Characterization of ferric and ferrous iron transport systems in *Vibrio cholerae*. *J. Bacteriol.* 188 6515–6523 10.1128/JB.00626-06 16952942 PMC1595488

[B237] Wyckoff E. E. Mey A. R. Payne S. M. (2007). Iron acquisition in *Vibrio cholerae*. *Biometals* 20 405–416 10.1007/s10534-006-9073-4 17216354

[B238] Xu H. S. Roberts N. Singleton F. L. Attwell R. W. Grimes D. J. Colwell R. R. (1982). Survival and viability of nonculturable *Escherichia coli* and *Vibrio cholerae* in the estuarine and marine environment. *Microb. Ecol.* 8 313–323 10.1007/BF02010671 24226049

[B239] Yamamoto S. Okujo N. Fujita Y. Saito M. Yoshida T. Shinoda S. (1993). Structures of two polyamine-containing catecholate siderophores from *Vibrio fluvialis*. *J. Biochem.* 113 538–544 8340347 10.1093/oxfordjournals.jbchem.a124079

[B240] Yamguchi T. Ikawa T. Nisizawa K. (1969). Pathway of mannitol formation during photosynthesis in brown algae. *Plant Cell Physiol.* 10 425–440

[B241] Yildiz F. H. (2008). Cyclic dimeric GMP signaling and regulation of surface-associated developmental programs. *J. Bacteriol.* 190 781–783 10.1128/JB.01852-07 18065536 PMC2223562

[B242] Yildiz F. H. Dolganov N. A. Schoolnik G. K. (2001). VpsR, a member of the response regulators of the two-component regulatory systems, is required for expression of vps biosynthesis genes and EPS(ETr)-associated phenotypes in *Vibrio cholerae* O1 El Tor. *J. Bacteriol.* 183 1716–1726 10.1128/JB.183.5.1716-1726.2001 11160103 PMC95057

[B243] Yildiz F. H. Liu X. S. Heydorn A. Schoolnik G. K. (2004). Molecular analysis of rugosity in a *Vibrio cholerae* O1 El Tor phase variant. *Mol. Microbiol.* 53 497–515 10.1111/j.1365-2958.2004.04154.x 15228530

[B244] Yildiz F. H. Schoolnik G. K. (1999). *Vibrio cholerae* O1 El Tor: identification of a gene cluster required for the rugose colony type, exopolysaccharide production, chlorine resistance, and biofilm formation. *Proc. Natl. Acad. Sci. U.S.A.* 96 4028–4033 10.1073/pnas.96.7.4028 10097157 PMC22414

[B245] Yildiz F. H. Visick K. L. (2009). *Vibrio* biofilms: so much the same yet so different. *Trends Microbiol.* 17 109–118 10.1016/j.tim.2008.12.004 19231189 PMC2729562

[B246] Ymele-Leki P. Houot L. Watnick P. I. (2013). Mannitol and the mannitol-specific enzyme IIB subunit activate *Vibrio cholerae* biofilm formation. *Appl. Environ. Microbiol.* 79 4675–4683 10.1128/AEM.01184-13 23728818 PMC3719523

[B247] Zhu J. Mekalanos J. J. (2003). Quorum sensing-dependent biofilms enhance colonization in *Vibrio cholerae*. *Dev. Cell* 5 647–656 10.1016/S1534-5807(03)00295-8 14536065

[B248] Zhu J. Miller M. B. Vance R. E. Dziejman M. Bassler B. L. Mekalanos J. J. (2002). Quorum-sensing regulators control virulence gene expression in *Vibrio cholerae*. *Proc. Natl. Acad. Sci. U.S.A.* 99 3129–3134 10.1073/pnas.052694299 11854465 PMC122484

